# Bacterial community associated to the pine wilt disease insect vectors *Monochamus galloprovincialis* and *Monochamus alternatus*

**DOI:** 10.1038/srep23908

**Published:** 2016-04-05

**Authors:** Marta Alves, Anabela Pereira, Patrícia Matos, Joana Henriques, Cláudia Vicente, Takuya Aikawa, Koichi Hasegawa, Francisco Nascimento, Manuel Mota, António Correia, Isabel Henriques

**Affiliations:** 1 Departamento de Biologia e Centro de Estudos do Ambiente e do Mar (CESAM), Universidade de Aveiro, Aveiro, 3810-193, Portugal; 2 Departamento de Biologia e Instituto de Biomedicina (iBiMED), Universidade de Aveiro, Aveiro, 3810-193, Portugal; 3 INIAV/Unidade Estratégica de Investigação e Serviços de Sistemas Agrários e Florestais e Sanidade Vegetal, Av. da República, Quinta do Marquês, Oeiras, 2780-157, Portugal; 4 NemaLab-ICAAM, Universidade de Évora, Núcleo da Mitra, Ap. 94, Évora, 7002-554, Portugal; 5 FFPRI - Forestry and Forest Products Research Institute, Tohuku, Japan; 6 Environmental Biology Department, Chubu University, Kasugai, Japan; 7 Dep. Ciências da Vida, EPCV, Universidade Lusófona de Humanidades e Tecnologias (ULHT), Av. Campo Grande 376, 1749-024 Lisboa, Portugal

## Abstract

*Monochamus* beetles are the dispersing vectors of the nematode *Bursaphelenchus xylophilus*, the causative agent of pine wilt disease (PWD). PWD inflicts significant damages in Eurasian pine forests. Symbiotic microorganisms have a large influence in insect survival. The aim of this study was to characterize the bacterial community associated to PWD vectors in Europe and East Asia using a culture-independent approach. Twenty-three *Monochamus galloprovincialis* were collected in Portugal (two different locations); twelve *Monochamus alternatus* were collected in Japan. DNA was extracted from the insects’ tracheas for 16S rDNA analysis through denaturing gradient gel electrophoresis and barcoded pyrosequencing. Enterobacteriales, Pseudomonadales, Vibrionales and Oceanospirilales were present in all samples. Enterobacteriaceae was represented by 52.2% of the total number of reads. Twenty-three OTUs were present in all locations. Significant differences existed between the microbiomes of the two insect species while for *M. galloprovincialis* there were no significant differences between samples from different Portuguese locations. This study presents a detailed description of the bacterial community colonizing the *Monochamus* insects’ tracheas. Several of the identified bacterial groups were described previously in association with pine trees and *B. xylophilus*, and their previously described functions suggest that they may play a relevant role in PWD.

*Monochamus* (Cerambycidae: Coleoptera) is a genus of sapro-xylophagous sawyer beetles^1^,^2^. In nature, they have a role in the processes of wood decomposition and nutrient cycling. Sawyer beetles can cause a significant loss in pine value due to the tunnels their larvae bore^3^. Yet, the major concern about *Monochamus* insects is their role as vectors of *Bursaphelenchus xylophilus* (Nematoda: Aphelenchoididae), the Pine Wilt Disease (PWD) causing agent^4^,^5^. This disease has been devastating pine trees since the beginning of the twentieth century with tremendous economic and environmental impacts^6^,^7^,^8^,^9^.

The tree-to-tree natural transmission of *B. xylophilus* is only possible through *Monochamus* beetles. The pinewood nematode (PWN) enters in its dispersal form into *Monochamus* when the beetle larvae molts to the late pupa^10^,^11^. *Bursaphelenchus xylophilus* concentrates mainly in the vectors’ tracheal system^12^, where it stays in a phoretic commensalism through insects’ development and emergence^9^. When the adult insect feeds, the PWN enter in a new host tree through the feeding wounds^13^. Once inside the host tree, *B. xylophilus* migrates through the vascular system and obstructs water conductance resulting in wilting of the pine host^8^.

Asia and Europe are the two most affected continents, *Monochamus alternatus* (Hope) being the most common vector in East Asia, and *Monochamus galloprovincialis* (Olivier) the most common vector in Europe^14^. In Japan, PWD was first reported in 1905^6^ and, during the first century of its occurrence, was responsible for the loss of 700 000 m^3^ of pine wood each year^8^. In Europe, the disease was first reported in Portugal in 1999 in the region of Setúbal Peninsula, south of Lisbon^15^. Despite the efforts of the Portuguese National Forestry and Quarantine Authorities, the disease has spread to the centre of mainland Portugal in 2008, and to Madeira Island, in 2009^7^.

The mechanism of the PWD is not completely elucidated. In 1980 a hypothesis that PWN-associated bacteria may be directly related with the disease development was suggested^16^. This hypothesis was based in the discovery of bacteria in *B. xylophilus* that produced a wilt toxin. Recent findings, using metagenomic approaches, suggest a role of PWN-associated bacteria in detoxification of xenobiotics inside conifers, suggesting a mutualistic symbiotic relationship between the nematode and its microbiome^17^.

The microbiome of the insect vector may also have a role in PWD. Studies focusing on disease vectoring efficiency demonstrated a large influence of symbiotic microorganisms in insect survival and developmental time^18^.The microbiome of *Monochamus* may influence the insect life cycle as well as the settling inside the host tree for oviposition, an essential step for the nematode acquisition and subsequent transportation. Due to the intimate association between the causative agent and its vector, it is admissible that PWN may acquire the insect’s bacterial community.

Only a few studies have characterized the bacteria associated to *Monochamus* spp. Most of these studies were based on culture-dependent methods not allowing an accurate approximation of the bacterial community composition^19^,^20^,^21^. To our knowledge, the only culture-independent study focused in *Monochamus* bacterial community was published by Vicente and colleagues (2013)^2^. The authors characterized the tracheal and abdominal bacterial community of eight nematode-free *M. galloprovincialis* specimens through the construction of 16S rRNA gene libraries and capillary sequencing. Proteobacteria, Firmicutes and Bacteroidetes were the dominant phyla, among which *Serratia*, *Bacillus* and *Janthinobacterium* were the most predominant genera^2^. Nevertheless, the applied methodology does not allow a detailed and deep characterization as, for instance, next-generation sequencing would do^22^.

A deeper understanding of the structure of the microbiome of the insect vector is necessary and might contribute to clarify the role of bacteria in PWD. Nowadays, pyrosequencing is a simple and cost-effective technology that enables us to analyze, for instance, the variability of the bacterial community between insect vectors from different locations and different species. The study presented here is the first to characterize the microbiome of PWD insect-vectors by applying deep 16S rRNA gene pyrosequencing. The bacterial community of *M. galloprovincialis* (the PWD insect-vector in Europe) and *M. alternatus* (the PWD insect-vector in Asia) was compared. Insects were collected in Portugal (in two different geographic locations) and Japan.

## Results

### General characterization of insect samples

A total of 35 insects (Table 1) were used in this study. Results from morphological and molecular methods for insect species identification were coherent. Therefore, the 23 insects collected in Portugal were identified as *M. galloprovincialis* and the 12 insects collected in Japan were identified as *M. alternatus*. Regarding insect gender, 12 of the *M. galloprovincialis* were females and 11 were males while 4 *M. alternatus* were females and 8 were males. *Bursaphelenchus xylophilus* was detected in 11 *M. alternatus* insects.

### DGGE analysis

DGGE analysis of the V3 hypervariable region of the 16S rRNA gene was used to compare the bacterial community structure between insects.

**Figure 1:**
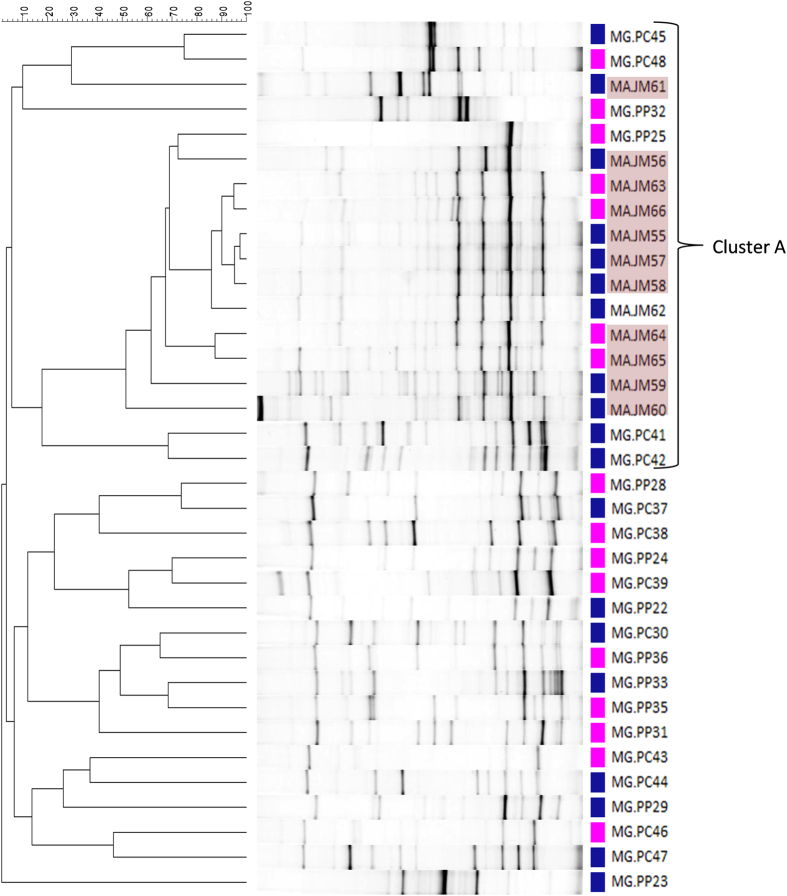
DGGE analysis of the V3 region from the 16S rRNA gene amplified from DNA of the trachea of *Monochamus* insects (MG – *Monochamus galloprovincialis*; MA – *Monochamus alternatus*) collected in Portugal (PC – Comporta or PM – Mortágua) and Japan (JH - Hikobe) respectively. The dendrogram of DGGE patterns was created using UPGMA method (group average method) and is based on Pearson similarity correlation (0.0%–100% similarity). Blue squares represent samples from males and pink squares represent samples from females. Samples in red background were positive for *Bursaphelenchus xylophilus*. Cluster A has *M. alternatus* samples.

Cluster analysis showed that *M. alternatus* samples grouped together (cluster A in Fig. 1) sharing more than 50% of similarity among them, with only one exception (sample MA.JH61). Statistical analysis confirmed that *M. galloprovincialis* profiles significantly differ from profiles obtained from *M. alternatus* (p < 0.05, PERMANOVA). Profiles did not cluster according to gender and Portuguese samples did not cluster according to sampling site (Mortágua and Comporta). In accordance, a PERMANOVA analysis showed no significant differences between profiles from male and female insects (p > 0.1) or between insects collected in Mortágua or Comporta (p > 0.05).

### Bacterial community composition

Seventeen samples were selected for pyrosequencing, 11 samples of *M. galloprovincialis* and 6 of *M. alternatus* (Table 2). Sample selection was based on DGGE analysis by picking several samples from the identified clusters.

A total of 185 275 raw reads were obtained that were filtered. In the dereplication step, 38 136 unique reads were identified. Singletons and chimeras were removed resulting in a final number of 228 OTUs. Twelve OTUs corresponded to eukaryotic DNA and were discarded from the analysis, resulting in a total of 216 OTUs and 159 280 high-quality reads (Table 2). Of the 216 OTUs, 210 were affiliated to the Bacteria domain while 6 could not be assigned (Supplementary Table S1). Two of the 210 assigned OTUs were only identified up to phylum level and 11 were only identified up to class level. From the 197 OTUs identified up to order level, 179 OTUs were identified at family level, 86 OTUs were identified at genus level and 16 OTUs were identified at species level (Supplementary Table S1).

The rarefaction curve for each insect sample tended to saturation (Figure S1), indicating that the OTUs detected were representative of the bacterial communities.

### *Monochamus galloprovincialis* bacterial community

A total number of 101 091 reads were obtained for *M. galloprovincialis* samples corresponding to 57 OTUs assigned to 5 different phyla: Proteobacteria, Firmicutes, Bacteroidetes, Acidobacteria and Actinobacteria. Proteobacteria was the dominant phylum, representing on average 98.4% of the reads in each sample with a standard deviation of ±2.0% (Supplementary Table S2). Firmicutes were present in 5 *M. galloprovincialis* samples and Bacteroidetes in 9 samples (out of 11). Firmicutes accounted on average for 0.7 ± 1.2% of the total reads in each sample and Bacteroidetes abundance averaged 0.7 ± 0.8%. Acidobacteria and Actinobacteria were each detected in one sample from Comporta, both with less than 1% of the total number of reads.

**Figure 2:**
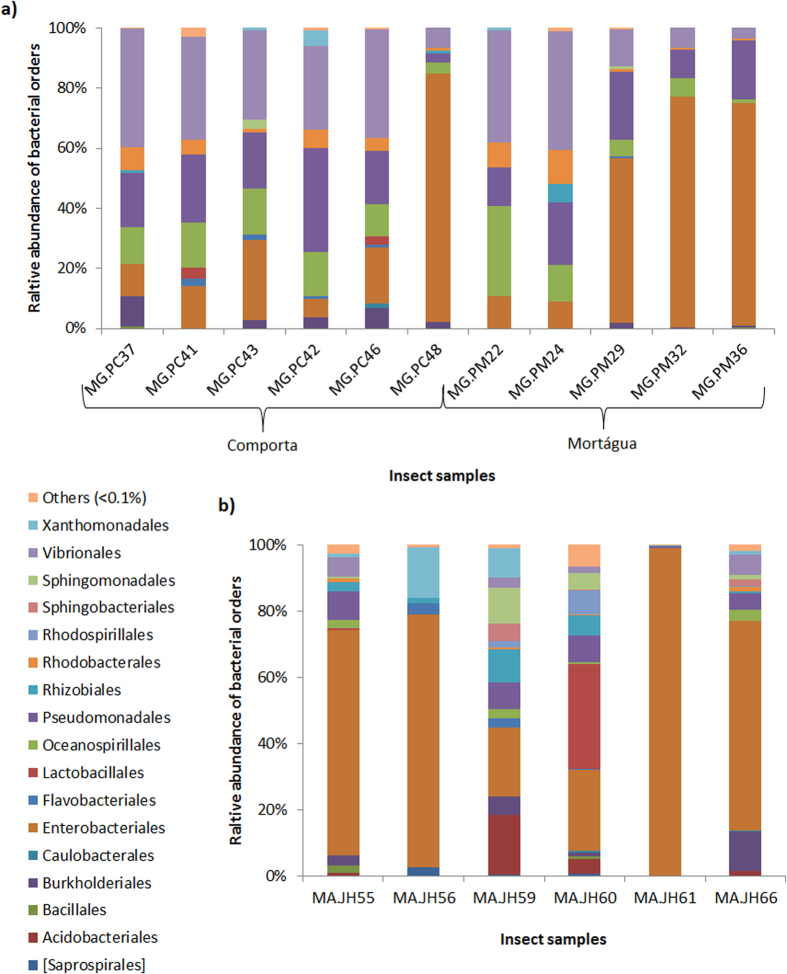
Relative abundance of the predominant bacterial orders for each insect sample (more than 0.1% of the total number of reads): (a) *Monochamus galloprovincialis* samples; (b) *Monochamus alternatus* samples.

Twenty one different bacterial orders were detected in *M. galloprovincialis* samples (Supplementary Table S3). The most represented orders were Vibrionales (24.9 ± 13.9%), Enterobacteriales (34.9 ± 29.3%), Pseudomonadales (18.2 ± 7.7%) and Oceanospirillales (11.6 ± 7.5%) representing altogether 91.5% of the total reads for the Portuguese host and detected in all samples. However, the relative abundance of each order differs among samples (Fig. 2a). For instance, from a total of 11 samples, Vibrionales was the most abundant order in 6, representing 36.2 ± 3.4% of the total reads in these 6 samples; Enterobacteriales was the most abundant order in 4 samples accounting for 72.1 ± 10.5%; Pseudomonadales was the most abundant order in one sample (MG.PC42) with 34.5% of the total reads (Supplementary Table S3).

**Figure 3:**
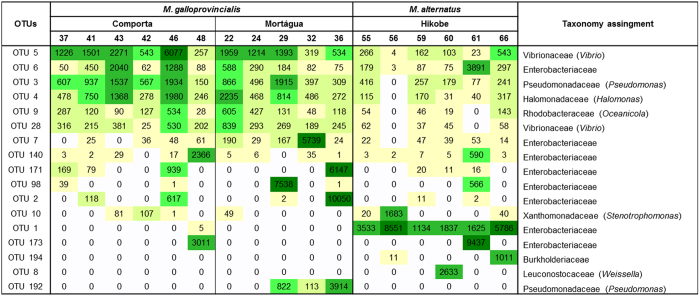
Heatmap for the OTUs that represent more than 10% of the total number of reads. The colour gradient goes from light yellow for the lower numbers of reads, to dark green for the higher numbers of reads. The numbers in the third top line represent the last two digits in the sample name (see Table 2).

The most abundant OTUs in our *M. galloprovincialis* dataset affiliated with the genus *Vibrio* (OTU 5, 17.1% of the total reads) and with the family Enterobacteriaceae (OTU 2, 10.7% of the total reads). From these, only OTU 5 (*Vibrio*) was present in all *M. galloprovincialis* samples. OTUs that were not only abundant (between 3 and 10% of the total reads) but also present in all *M. galloprovincialis* samples, affiliated with *Pseudomonas* (OTU 3), *Halomonas* (OTU 4), *Oceanicola* (OTU 9), *Vibrio* (OTU 28) and with Enterobacteriaceae family (OTU 6) (Fig. 3). PERMANOVA analysis of the OTU abundances confirmed that there were no significant differences between the bacterial communities of insects of the two sampling sites (Comporta and Mortágua) (p > 0.05).

Shannon diversity index (H) and Pielou equitability index (E) were calculated for each sample (Table 2). Values of H ranged from 1.24 to 2.30 for *M. galloprovincialis*. Values of E ranged from 0.42 and 0.77. The lower values of H and E show an irregular taxa distribution. Richness across *M. galloprovincialis* samples ranged from 12 to 24 OTUs. There were no significant differences in the indexes among samples from Comporta and Mortágua (P > 0.05, Student’s *t*-tests for S and H indexes and Mann-Whitney test for J index).

### *Monochamus alternatus* bacterial community

A total number of 58 189 reads were obtained for *M. alternatus* samples corresponding to 199 OTUs assigned to 11 different phyla. Proteobacteria was the most abundant phylum in all Japanese samples representing on average 85.4 ± 15.8% (Supplementary Table S2). Proteobacteria was followed by Firmicutes (6.0 ± 11.9%), Acidobacteria (4.5 ± 6.5%) and Bacteroidetes (2.9 ± 3.1%). Proteobacteria, Acidobacteria and Bacteroidetes were present in all *M. alternatus* samples. Each of the remaining 7 bacterial phyla represented less than 1% of the total reads.

**Figure 4:**
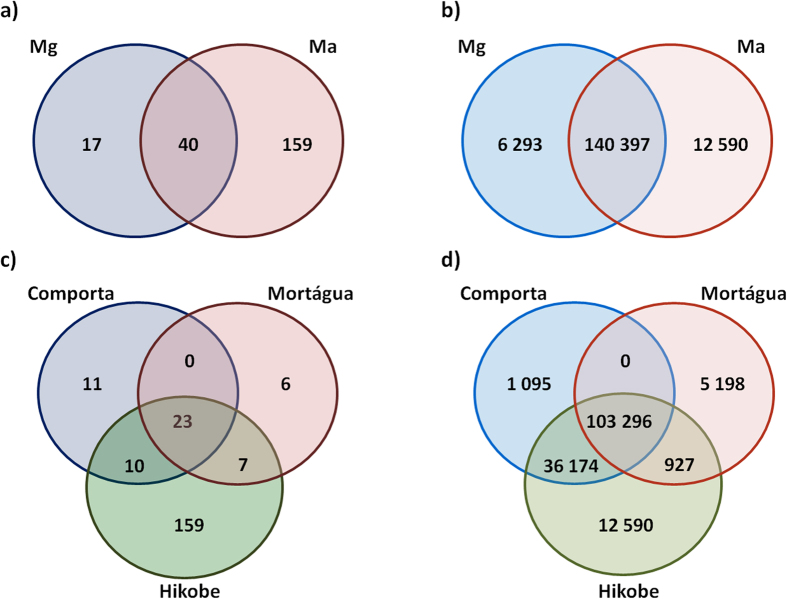
Venn diagrams representing the number of (a) shared and unique OTUs and (b) shared and unique reads between *Monochamus galloprovincialis* (Mg) and *Monochamus alternatus* and the number of (c) shared and unique OTUs and (d) shared and unique reads between sampling locations (Comporta, Mortágua and Hikobe).

Thirty-seven bacterial orders were identified in *M. alternatus* (Supplementary Table S3). Enterobacteriales was the most abundant order in all samples (58.6 ± 27.7%) except for sample MA.JM60 (Supplementary Table S4 and Fig. 4c). Lactobacillales was the most abundant order in MA.JM60 (31.8% of the reads). The second most abundant order was different in each sample (Table 2b). Nevertheless, the bacterial orders represented with more than 2% of the total reads were: Pseudomonadales (5.1 ± 3.6%), Rhizobiales (3.4 ± 3.5%), Sphingomonadales (2.9 ± 3.9%), Vibrionales (2.9 ± 2.5%), Flavobacteriales (1.1 ± 1.4%) and Xanthomonadales (4.3 ± 5.7%). All mentioned orders, except Xanthomonadales, were present in all *M. alternatus* samples.

The most abundant OTUs in the *M. alternatus* dataset and present in all *M. alternatus* samples affiliated with Enterobacteriaceae family (OTU 1 and OTU 6).

Concerning alpha diversity for *M. alternatus* samples, values of diversity (H) ranged from 0.86 to 3.40, values of equitability (E) ranged from 0.26 and 0.80 and values of richness (S) ranged from 27 and 109 (Table 2).

### Bacterial community of *Monochamus galloprovincialis* and *M. alternatus*: common and unique features

The tracheal bacterial communities hosted by *M. alternatus* and *M. galloprovincialis* display some common traces. Proteobacteria, Firmicutes and Bacteroidetes were the most abundant phyla in both host species. The bacterial orders Enterobacteriales, Pseudomonadales, Vibrionales and Oceanospirilalles were present in all insect samples. Enterobacteriaceae was the most abundant family in both species, representing 52.2% of the total number of reads (34.91 ± 29.27% for *M. galloprovincialis* and 58.58 ± 27.74% for *M. alternatus*). Forty OTUs, corresponding to 88.1% of reads, were detected in both species of insects (Fig. 4a,b). From these, 23 OTUs were present in insects from all sampling locations (Fig. 4c,d), corresponding to 64.9% of the total number of reads, affiliating with the classes: Gammaproteobacteria (18 OTUs), Alphaproteobacteria (3 OTUs), Betaproteobacteria (1 OTU) and Flavobacteria (1 OTU). The most represented of these OTUs (more than 10% of the total reads) belong to the families Enterobacteriaceae (6 OTUs), Vibrionaceae (2 OTUs belonging to the genus *Vibrio*), Pseudomonadaceae (2 OTUs), Halomonadaceae (1 OTU belonging to the genus *Halomonas*), Rhodobacteraceae (1 OTU belonging to the genus *Oceanicola*) and Xanthomonadaceae (1 OTU belonging to the genus *Stenotrophomonas*) (Fig. 3).

**Figure 5:**
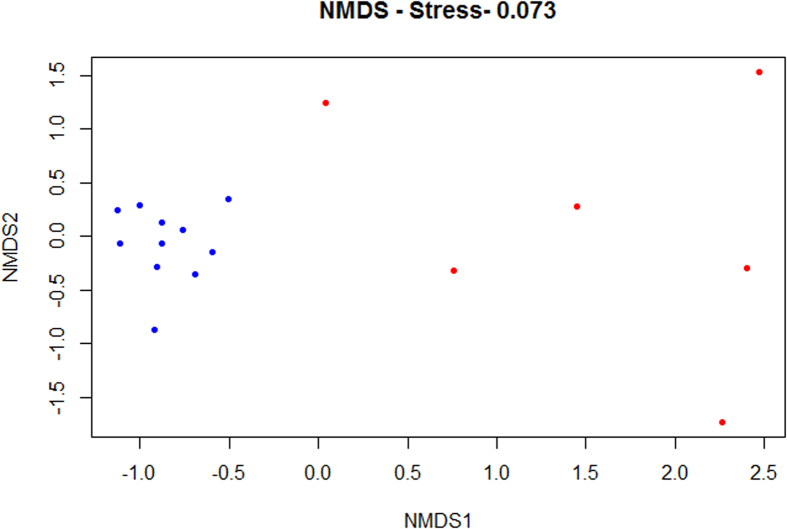
Bacterial community compositional structure in *Monochamus galloprovincialis* samples (blue) and *Monochamus alternatus* samples (red) indicated by non-metric multi-dimensional scaling (NMDS) with a stress value of 0.073, using Bray-Curtis distance.

Despite the resemblances of the bacterial communities of both insect species, through NMDS analysis (Fig. 5) it was possible to observe a separation between *M. galloprovincialis* samples and the ones from *M. alternatus*. The bacterial community composition was significantly different between both host species (P < 0.05, PERMANOVA).

Tracheal bacterial communities from *M. alternatus* included 16 more bacterial orders than those from *M. galloprovincialis*. Acidobacteriales was detected in all *M. alternatus* samples, being the sixth most abundant bacterial order for this host (3%), but was not present in any of the *M. galloprovincialis* samples. Lactobacillales and Xanthomonadales abundance was higher in *M. alternatus* (5% and 4%) than in *M. galloprovincialis* (1% and 0.2%). Inversely, the orders Vibrionales, Pseudomonadales, Oceanospirillales and Rhodobacterales were frequently detected but in higher abundances in *M. galloprovincialis* (14.7 ± 11.8%, average for the 4 bacterial orders) than in *M. alternatus* (2.6 ± 2.9%).

Differences could also be observed regarding the abundance of dominant OTUs (Fig. 3). OTU 1 (Enterobacteriaceae) was represented by a higher number of reads (6.4 ± 4.6% average of the total reads) for the *M. alternatus* samples than for *M. galloprovincialis* (0.005%); OTUs 3, 4, 9 and 28 were more represented in *M. galloprovincialis* (2.3 ± 1.4%, average of reads per sample) than in *M. alternatus* (0.7 ± 0.5%).

The number of OTUs was much higher in *M. alternatus* (199) than in *M. galloprovincialis* (57) with significant differences in species richness between insects species (P < 0.05 Mann-Whitney test). There were no significant differences among the diversity and equitability indexes between the tracheal bacterial communities from *M. alternatus* and *M. galloprovincialis* (p > 0.05, Student’s t-test).

## Discussion

The goal of this study was to characterize the bacterial community colonizing the trachea of *M. galloprovincialis* and *M. alternatus,* two insect vectors of *B. xylophilus*, the causative agent of PWD^5^. The trachea of the insect vector is the preferential organ for nematode lodging^12^.

The role of bacteria in PWD is a controversial subject^23^ but nowadays the microbiome is acknowledged as very important to host fitness and adaptation. In insect-plant interaction, microbes are described as crucial mediators, especially for herbivorous insects^24^. Thus, characterizing the microbiome of the PWD insect-vectors is an important step to better understand the disease mechanism and the role of bacteria in the pathogenic process. Considering the nematode life cycle and its relation with the insect, it is reasonable to place the focus on the microbiome of the trachea^9^.

To deeply characterize the bacterial community of the insects’ tracheas, a culture-independent approach was used, combining DGGE analysis and barcoded pyrosequencing. DGGE analysis is described as a cost- and time-effective technique, suitable to evaluate compositional variation of complex bacterial communities^25^. This technique has been used in combination with massive parallel sequencing in many studies, allowing a global view of the community structure of a high number of samples and the selection of a smaller set for deep characterization^26^.

The results from this study show that the bacterial community of both *Monochamus* species is dominated by Proteobacteria, mostly represented by Gammaproteobacteria, as described for other herbivorous insects^25^. Gammaproteobacteria has also been described as the dominant class in the only culture-independent study of the microbiome of the genus *Monochamus*^2^.

In the current study, the main bacterial orders detected in the two insect vectors of *B. xylophilus* were: Enterobacteriales, Pseudomonadales, Vibrionales and Oceanospirilalles. Two OTUs (affiliated to *Vibrio* and Enterobacteriaceae) were present in all insect samples representing more than 10% of the total number of reads.

Vibrionales and Oceanospirilalles were for the first time reported in association with *B. xylophilus* insect-vectors. The most represented genera for these orders were *Vibrio* and *Halomonas*, respectively. Both *Halomonas* spp. and *Vibrio* spp. have been reported to have cellulolytic activity^27^,^28^. *Vibrio* species were also detected in the hindgut of *Balanogastris kolae,* an herbivorous insect pest, with proteolytic and amylolytic activity^29^. This ability for degradation of cellulose, proteins and starch appears to be important for herbivorous insects as the ones in this study.

Both Enterobacteriales and Pseudomonadales orders have already been described in *M. galloprovincialis* and *M. alternatus*, in culture-independent and dependent methods respectively^2^,^20^.

The mentioned bacterial groups may have a role in the insect life cycle, in its different stages, affecting PWD spreading. Egg- and larvae-associated microbiome, which may be vertically transmitted from the adult female, is described as very important for their survival^30^. For instance, *Pseudomonas*, the most represented genus of Pseudomonadales found in this study, was reported as having a role in insect egg protection against attack by other microbes^31^.

As suggested for *B. xylophilus*^19^, the insect vector microbiome might also play a role in the defence against harmful compounds produced by the plant. *Pseudomonas* may help both through supressing plant defences as described by Chung and colleagues (2013)^32^ or through chemical detoxification, once this genus was reported to survive the stress of α-pinene (an organic compound found in coniferous trees), being able to use this compound as a carbon source^17^. Members of the same genus were also detected in other studies as part of consortia that protect insects from polluted environments^33^ and in other longhorn beetles putatively implicated in the degradation of aromatic hydrocarbons^34^.

Other bacterial groups detected in this study are known to display metabolic capacities that might help the survival of *B. xylophilus* and its insect vector during pine infection. *Vibrio* spp. are known to play a role in processing pollutants, including aromatic compounds^35^,^36^. *Stenotrophomonas*, represented by OTU 10 present in all sampling locations, was also reported in previous studies to survive the stress of α-pinene being able to degrade this compound^17^.

Besides *Monochamus* spp., Enterobacteriales and Pseudomonadales were also previously described in *B. xylophilus* isolated from different pine species (including *P. pinaster* and *P. densiflora)* and also in *Pinus pinaster* trees^2^,^37^,^38^,^39^. The presence of the same orders in the three organisms that take part in the cycle of the PWD suggests that these orders might have a role in the disease mechanism. Some groups of these bacterial orders are considered quarantine pests (that may represent a risk with potential economic importance)^40^, mostly for their toxin-producing ability. Members of Enterobacteriales were described as producers of cell wall degrading enzymes (cellulases, peptidases and chitinases) and as highly resistant to H_2_O_2_ (the most predominant reactive oxygen species, ROS, produced by the plant-basal defences against biological stresses)^41^,^42^. These abilities may be relevant in the PWD mechanism.

The presence of the same bacterial groups in the three PWD organisms may also be related to the transference of bacteria between these organisms. For example, the origin of *B. xylophilus* bacterial community is unknown and in previous studies it has been suggested that it might be transmitted from the insect-vector to the nematode^2^,^23^ but more studies must be conducted to confirm this theory.

Both methods used in this study, DGGE and pyrosequencing, revealed differences between the trachea microbiomes of *M. alternatus* and *M. galloprovincialis,* suggesting the existence of a species-specific microbiome. Vibrionaceae, Pseudomonadaceae and Halomonadaceae are highly abundant in *M. galloprovincialis* samples having a minor importance in the communities from *M. alternatus*. Inversely, the order Xanthomonadaceae is more abundant in *M. alternatus* samples and the family Acidobacteriaceae is present only in *M. alternatus* samples, being the third most abundant family in this species. In general, pyrosequencing revealed higher diversity in *M. alternatus* than in *M. galloprovincialis*. The existence of a species-specific microbiome was also reported for other insect species^43^. Bacterial communities might have to adapt to differences in morphology and life cycle between species with the consequent variations in the community structure. Also, these two insect species do not co-exist in the same country, having different tree hosts. As reported for *B. xylophilus*^44^, the bacterial community associated to *Monochamus* spp. may also differ according with the environmental factors and pine species host. According to the Köppen and Geiger classification^45^, Hikobe (the sampling site in Japan) is Dsa (D - Cold; s - dry summer; a - hot summer) with cold temperate weather and both Portuguese locations are Csa (C – Temperate) with warm temperate weather. Also, different pine species from different locations have differences in their physiology that would affect their microbiota and consequently the insects they host^46^. Thus, these *Monochamus* species must be adapted to their endemic regions and their tree hosts, with differences in their bacterial community to favour that adaptation.

The hypothesis that the differences between the bacterial communities from the two insect species here studied are due to the unbalanced proportion of insects with and without *B. xylophilus* in their tracheas was considered: in all pyrosequenced *M. galloprovincialis* the nematode was absent and all *M. alternatus* samples carried *B. xylophilus*. Nevertheless, the *B. xylophilus* was not detected by visual inspection of the trachea either in *M. galloprovincialis* and *M. alternatus*, and was only detected when a highly sensitive PCR-based method was used. Positive results may be due to the presence of either a residual number of *B. xylophilus* or *B. xylophilus* free DNA. Thus it is not probable that the observed differences are attributable to the presence/absence of *B. xylophilus*. Besides, the DGGE profile from *M. alternatus* without *B. xylophilus* (i.e. MA.JH62; Fig. 1) shared more than 80% similarity with *M. alternatus* samples with *B. xylophilus*, also suggesting that the presence of *B. xylophilus* had minor or no influence on the insect bacterial community. In addition, a main difference between *M. alternatus* and *M. galloprovincialis* microbiomes was the dominance of the orders Acidobacteriales, Xanthomonadales and Lactobacillales in *M. alternatus* bacterial community. Despite the existence of several culture-independent studies describing the bacterial community associated to *B. xylophilus*^17^,^47^, members of the orders Acidobacteriales and Lactobacillales were not reported so far in association with the nematode. Thus, the abundance of these orders in the microbiome of *M. alternatus* does not seem to be related to the presence of *B. xylophilus*.

Concerning *M. galloprovincialis* samples, there were no significant differences between the microbiome of insects collected in Comporta and Mortágua, suggesting that sampling location had no influence in *M. galloprovincialis* microbiome. This stability in the microbiome may suggest relevance in the disease mechanism. Comporta and Mortágua are both temperate regions and the same *Pinus* species was collected (*Pinus pinaster*). However, there were site-specific OTUs, like OTU 192 affiliated with *Pseudomonas*, which was present only in Mortágua samples in high abundance. These specificities may result from the bacterial community adaptation to the local environment; despite the mentioned clime resemblances, sampling locations have differences in altitude (locations differ in 94 m of altitude) and precipitation regimens.

## Conclusions

This study suggests that the microbiome of *Monochamus* tracheae is species-specific and independent of the insect gender and sampling location. More studies will be necessary to determine both the effect of the presence of *B. xylophilus* in the insect microbiome and the effect of the host tree. Several bacterial groups detected here have been described in other studies in association with the other two PWD organisms (*Pinus pinaster* trees and *B. xylophilus*) suggesting that these bacteria might play a role in the disease development. Some taxa might be involved in processes of detoxification of harmful compounds, others might produce toxins, both helping the tree invasion. The insect microbiome characterization is important not only to better understand the role of bacteria in PWD but also for the elaboration of bio-control strategies.

## Methods

### Sampling

Symptomatic adult trees with symptoms from class IV according with Proença and colleagues (2010)^38^ - withered trees with more than 80% of brown needles - were selected in the spring (April and May) of 2013 in locations known to be affected by PWD^48^,^49^: *Pinus pinaster* trees were selected in Portugal from Mortágua (40°24′17.2″N 8°13′32.7″W) and Comporta (Herdade da Comporta, 38°22′48.67″N/8°47′25.00″W), central and south regions of Portugal respectively, and *Pinus densiflora* trees in Japan from Hikobe (Shiwa District, Iwate prefecture, 34°27′.00″N/133°46′.00″E). Specifically, log sections were sampled, after being inspected for the presence of *Monochamus* entry-points. Logs were placed in black plastic containers covered at the top with semi-transparent cloth meshes and kept inside a greenhouse. The wood material from each tree was divided according to its origin and kept isolated. All insects used for this study were caught alive after emergence from the tree logs. Those morphologically identified as *M. galloprovincialis* and *M. alternatus* were sexed.

As described by Kobayashi and colleagues (1984)^12^, *Bursaphelenchus xylophilus* concentrates mainly in the vectors’ tracheal system. For that reason, the bacterial community associated to insects’ tracheae may play a role in the PWD mechanism. Thus, each insect was surface sterilized in a 70% ethanol solution for 1 minute. After rinsing in sterile distilled water, insects were dissected under a binocular scope (Wild Heerbrugg, Switzerland) for trachea extraction.

### DNA extraction from insect tracheae, molecular analysis for *Monochamus* species confirmation and *B. xylophilus* detection

Tracheae were placed in 40 μl of sterile 1× phosphate-buffered saline (PBS) and total DNA extraction was performed using PowerSoil^®^ DNA Isolation Kit according to the manufacturer’s instructions (MOBIO, CA, USA). Insect morphological identification was confirmed by amplification and sequencing of cytochrome c oxidase I (COI) gene using the primers C1-J-2183a (5′-CAACAYTTATTTTGATTTTTTGG-3′) and TL2-N-3014 (5′-TCCAATGCACTAATCTGCCATATTA-3′)^50^. The amplification conditions were as follows: an initial denaturation step at 95 °C for 10 min, followed by the three step amplification with 30 cycles of denaturation at 95 °C for 30 s, annealing at 52 °C for 30 s and extension at 72 °C for 70 s, and a final extension step at 72 °C for 10 min. The reaction mixtures (25 μL) consisted of 6.25 μL NZYTaq 2× Green Master Mix (2.5 mM MgCl_2_; 200 μM dNTPs; 0.2 U/μL DNA polymerase) (NZYTech, Portugal), 16.25 μL of ultrapure water, 0.03 μM and 50–100 ng of purified DNA of template DNA. In this and subsequent PCR experiments, negative controls were used replacing the template DNA with sterile dH_2_O. PCR reactions were conducted in a Bio-Rad Thermal Cycler (Bio-Rad Laboratories, CA, USA). Products were analysed by electrophoresis on a 1.5% agarose gel and stained with ethidium bromide.

Amplicons were purified with the JetQuick^®^ PCR Product Purification Spin Kit (Genomed, Germany) following the manufacturer’s instructions and used as template in the sequencing reactions carried out by the company GATC (Germany) with the primer C1-J-2195 (5′-TTGATTTTTTGGTCATCCAGAAGT-3′)^50^.

All samples were screened for *B. xylophilus* by PCR amplification of the species specific *MspI* satDNA according with the European and Mediterranean Plant Protection Organization^51^ instructions using the primers and conditions previously described^52^.

### Denaturing Gradient Gel Electrophoresis (DGGE) analysis

A nested PCR technique was applied to amplify the V3 region of the 16S rRNA gene in order to increase sensitivity. Total DNA extracted as described above was used as substrate. First, the 16S rRNA gene was amplified using the universal primers 27F (5′-AGAGTTTGATCCTGGCTCAG-3′) and 1492R (5′-GGTTACCTTGTTACGACTT-3′)^53^. The amplification conditions and reaction mixtures consisted of an initial denaturation step (94 °C for 3 min), followed by 30 amplification cycles comprising the following steps: denaturation (94 °C for 1 min), annealing (52 °C for 1 min) and extension (72 °C for 2 min), and a final extension step (72 °C for 10 min). The reaction mixtures were performed as described in Henriques *et al.*^25^. The second PCR was conducted using 1 μL of the first PCR product as template and the same reagents and volumes except for the primers. The V3 region of bacterial 16S rRNA gene was the target of the second amplification using primers and conditions as described in Henriques *et al.*^25^. Positive and negative controls were used in both PCRs, replacing the template DNA with DNA from *Escherichia coli* ATCC 25922 and with sterile dH_2_O respectively.

PCR products were applied onto 8% polyacrylamide (37.5:1, acrylamide/bisacrylamide) gel with linear denaturing gradient ranging from 30% to 65% (100% corresponds to 7 M Urea and 40% formamide). Electrophoresis was conducted in a DCode System (Bio-Rad) as described in Henriques *et al.*^25^.

Gel images were acquired using a Molecular Imager^®^ Gel Doc^TM^ XR+ System (Bio Rad Laboratories, Hercules, California, USA). Every DGGE gel contained, at its ends, two lanes with a standard of eight bands for internal and external normalization and as an indication of the quality of the analysis^25^. Replication of the PCR and DGGE steps was performed to confirm the consistency of the profiles.

DGGE patterns were analysed using Bionumerics Software (Applied Maths, Belgium). Cluster analysis of DGGE profiles was performed using the UPGMA method (group average method) applying Pearson correlation coefficient.

### Barcoded 454 Pyrosequencing

Samples were prepared for barcoded 454 pyrosequencing by nested-PCR amplification of the 16S rRNA gene. The first amplification reaction was done as described for DGGE analysis; the second PCR targeted the V3–4 hypervariable region of the 16S rRNA gene, using the forward primer 5′– ACTCCTACGGGAGGCAG-3′ and the reverse primer 5′– TACNVRRGTHTCTAATYC -3′^54^. The second set of primers was ligated to the Roche-454 A and B Titanium sequencing adapters and to an eight-base barcode sequence. For the second amplification fragments were amplified in 40 μL reactions with Advantage Taq (Clontech) using 0.2 μM of each primer, 0.2 mM dNTPs, 1× polymerase mix and 6% DMSO. PCR conditions were 94 °C for 4 min, followed by 25 cycles of 94 °C for 30 s, 44 °C for 45 s and 68 °C for 60 s and a final elongation step at 68 °C for 10 min. The amplicons were quantified by fluorometry with PicoGreen (Invitrogen, CA, USA), pooled at equimolar concentrations and sequenced in the A direction with GS 454 FLX Titanium chemistry, according to manufacturer’s instructions (Roche, 454 Life Sciences, Brandford, CT, USA) at Biocant (Cantanhede, Portugal). Each DNA sequence was afterwards traced back to its original sample through barcode analysis.

Sequences were processed using both UPARSE^55^ and QIIME^56^ pipelines on a computer using the Linux© operating system.

In the UPARSE workflow, barcodes were striped and reads were quality filtered to a maximum expected error of 1.0 and trimmed to 400 bp. Sequences were dereplicated (identical reads were merged) as described by Edgar (2015)^55^, generating a file containing a set of unique read sequences each marked with and integer value indicating its abundance. After singletons removal, Operational Taxonomic Units (OTUs) were defined at 97% similarity applying the UPARSE-OTU algorithm (that simultaneously identifies and discards chimeras) to the set of unique read sequences. Chimera check was done a second time running uchime_ref on the OTU sequences generated. Taxonomy assignment was made through QIIME using Uclust as assignment method and Greengenes reference databases. Concerning alpha-diversity, rarefaction plot for the observed species for each sample was made using QIIME scripts; richness index, S, Shannon index of diversity, H^57^ and the equitability index, E^58^ were calculated using PRIMER software^59^ for each sample as follows:


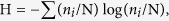



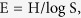


*n*_*i*_ is the OTU abundance, S is the number OTUs (used to indicate the number of species) and N is the sum of all reads for a given sample (used as estimates of species abundance)^60^.

Previously to statistical and beta-diversity analysis, sequences were first rarefied to the lowest number of reads in the samples using QIIME script single_rarefaction.py.

All sequences obtained are available in the NCBI platform with the accession numbers SRX1246548, SRX1249956.

### Statistical Analysis

Statistical analysis was used to determine if any of the following factors had significant influence (P < 0.05) in the composition of bacterial communities: sex of insects, their species, presence of *B. xylophilus* and sampling sites. Statistical significance for each factor was evaluated through PERMANOVA based on 999 permutations using PRIMER v6 software^59^. Both DGGE and pyrosequencing dataset were transformed using the log(x + 1) transformation and PERMANOVA was performed on Bray-Curtis distance matrixes constructed from the transformed DGGE table and from the rarefied and transformed OTU abundance table.

Non-metric Multidimensional Scaling (NMDS) was applied to the rarefied and transformed (log(x + 1)) OTU abundance table. This analysis was based on Bray-Curtis distance and computed using R Software (www.r-project.org).

Statistical significance of variance in indexes (S, H and E) was evaluated with Student’s *t*-tests when data had a normal distribution (checked using the Shapiro-Wilk test). For non-normally distributed data, the Mann-Whitney test was applied. Shapiro-Wilk, Student’s t and Mann-Whitney tests were computed using R Software (www.r-project.org).

## Additional Information

**How to cite this article**: Alves, M. *et al.* Bacterial community associated to the pine wilt disease insect vectors *Monochamus galloprovincialis* and *Monochamus alternatus. Sci. Rep.* **6**, 23908; doi: 10.1038/srep23908 (2016).

## Supplementary Material

Supplementary Information

## Figures and Tables

**Table 1:** Insects used in this study according to species, geographic origin, gender and presence/absence of *Bursaphelenchus xylophilus*.

Species	Location (Country)	NrInsects	*B. xylophilus*carriers	Nrfemales	Nrmales
*Monochamus alternatus*	Hikobe (Japan)	12	11	4	8
*Monochamus galloprovincialis*	Mortágua (Portugal)	12	0	7	5
	Comporta (Portugal)	11	0	5	6
**Total**	3 (2)	35	11	16	19

**Table 2:** Total number of reads obtained for each sample selected for pyrosequencing and the respective indexes of richness (S, number of OTUs), equitability (E, Pielou index) and diversity (H, Shannon index).

Species	Location	Samples	Reads	S	E	H
*Monochamus galloprovincialis*	Comporta	MG.PC37	3905	18	0.74	2.13
		MG.PC41	4968	22	0.70	2.18
		MG.PC43	8921	17	0.71	2.00
		MG.PC42	2042	19	0.72	2.12
		MG.PC46	18345	23	0.73	2.30
		MG.PC48	6951	19	0.54	1.60
	Mortágua	MG.PM22	7443	12	0.73	1.81
		MG.PM24	3799	15	0.77	2.10
		MG.PM29	14657	23	0.57	1.80
		MG.PM32	7989	19	0.42	1.24
		MG.PM36	22071	24	0.45	1.44
*Monochamus alternatus*	Hikobe	MA.JH55	5504	63	0.42	1.73
		MA.JH56	11212	27	0.26	0.86
		MA.JH59	6308	73	0.80	3.45
		MA.JH60	8340	109	0.60	2.80
		MA.JH61	17156	41	0.37	1.38
		MA.JH66	9669	43	0.47	1.77
		**Total**	**159280**	**216**		

## References

[b1] Linsley E. G. Ecology of Cerambycidae. Annu. Rev.Entomol. 4, 99–138 (1959).

[b2] Vicente C. S. *et al.* Characterization of bacterial communities associated with the pine sawyer beetle *Monochamus galloprovincialis*, the insect vector of the pinewood nematode *Bursaphelenchus xylophilus*. FEMS Microbiol. Lett. 347, 130–139 (2013a). 23927049 10.1111/1574-6968.12232

[b3] Bélanger S. *et al.* Effect of temperature and tree species on damage progression caused by whitespotted sawyer (Coleoptera: Cerambycidae) larvae in recently burned logs. J. Econ. Entomol. 106, 1331–1338 (2013). 23865199 10.1603/ec12372

[b4] Mamiya Y. & Enda N. Transmission of *Bursaphelenchus lignicolus* (Nematoda: Aphelenchoididae) by *Monochamus alternatus* (Coleoptera: Cerambycidae). Nematologica 18, 159–162 (1972).

[b5] Futai K. Pine Wood Nematode, Bursaphelenchus xylophilus. Annu. Rev. Phytopathol. 51, 61–83 (2013). 23663004 10.1146/annurev-phyto-081211-172910

[b6] Mamiya Y. History of pine wilt disease in Japan. J. Nematol. 20, 219–226 (1988). 19290205 PMC2618808

[b7] Mota M. & Vieira P. Pine Wilt Disease: A worldwide treath to forest ecosystems. (Springer, Netherlands, 2008).

[b8] Vicente C., Espada M., Vieira P. & Mota M. Pine Wilt Disease: a threat to European forestry. Eur. J. Plant Pathol. 133, 89–99 (2012a).

[b9] Zhao L., Mota M., Vieira P., Butcher R. A. & Sun J. Interspecific communication between pinewood nematode, its insect vector, and associated microbes. Trends Parasitol. 30, 299–308 (2014). 24810363 10.1016/j.pt.2014.04.007

[b10] Linit M. J. Nematode-vector relationships in the Pine Wilt Disease. J. Nematol. 20, 227–235 (1988). 19290206 PMC2618795

[b11] Zhao L. *et al.* Chemical signals synchronize the life cycles of a plant-parasitic nematode and its vector beetle. Curr. Biol. 23, 2038–2043 (2013). 24120638 10.1016/j.cub.2013.08.041

[b12] Kobayashi F., Yamane A. & Ikeda T. The Japanese pine sawyer beetle as the vector of pine wilt disease. Annu. Rev. Entomol. 29, 115–135 (1984).

[b13] Jones J. T., Moens M., Mota M., Li H. & Kikuchi T. *Bursaphelenchus xylophilus*: opportunities in comparative genomics and molecular host–parasite interactions. Mol. Plant. Pathol. 9, 357–368 (2008). 18705876 10.1111/j.1364-3703.2007.00461.x PMC6640334

[b14] Pimentel C. S., Ayres M. P., Vallery E., Young C. & Streett D. A. Geographical variation in seasonality and life history of pine sawyer beetles *Monochamus* spp: its relationship with phoresy by the pinewood nematode *Bursaphelenchus xylophilus*. Agric. For. Entomol. 16, 196–206 (2014).

[b15] Mota M. *et al.* First report of *Bursaphelenchus xylophilus* in Portugal and in Europe. Nematology. 1, 727–734 (1999).

[b16] Oku H., Shiraishi T., Ouchi S., Kurozumi S. & Ohta H. Pine wilt toxin, the metabolite of a bacterium associated with a nematode. Naturwissenschaften. 67, 198–199 (1980).

[b17] Cheng X. Y. *et al.* Metagenomic analysis of the pinewood nematode microbiome reveals a symbiotic relationship critical for xenobiotics degradation. Sci. Rep. 3, 1869 (2013). 23694939 10.1038/srep01869 PMC3660777

[b18] Ricci I. *et al.* Symbiotic control of mosquito borne disease. Pathog. Glob. Health. 106, 380–385 (2012). 23265608 10.1179/2047773212Y.0000000051 PMC4001619

[b19] Ma L. J., Zhang L. Q., Lin H. P. & Mao S. F. Investigation of pathogens of *Monochamus alternatus* in East China and virulence. Chin. J. Biol. Control. 25, 220–224 (2009).

[b20] Park D. S. A culture-based study of the bacterial communities within the guts of nine longicorn beetle species and their exo-enzyme producing properties for degrading xylan and pectin. J. Microb. 45, 394–401 (2007). 17978798

[b21] Soper R. S. & Olson R. E. Survey of biota associated with *Monochamus* (Coleoptera: Cerambycidae) in Maine. Can. Entomol. 95, 83–95 (1963).

[b22] Nikolaki S. & Tsiamis G. Microbial diversity in the era of omic technologies. BioMed Res. Int. 2013, e958719 (2013). 10.1155/2013/958719 PMC3821902 24260747

[b23] Nascimento F. X., Hasegawa K., Mota M. & Vicente C. S. L. Bacterial role in pine wilt disease development – review and future perspectives. Environ. Microbiol. Rep. 7, 51–63 (2015). 25139220 10.1111/1758-2229.12202

[b24] Hansen A. K. & Moran N. A. The impact of microbial symbionts on host plant utilization by herbivorous insects. Mol. Ecol. 23, 1473–1496 (2014). 23952067 10.1111/mec.12421

[b25] Henriques I. *et al.* Seasonal and spatial variability of free-living bacterial community composition along an estuarine gradient (Ria de Aveiro, Portugal). Estuarine Coastal Shelf Sci. 68, 139–48 (2006).

[b26] Cleary D. F. R., Smalla K., Mendonça-Hagler L. C. S. & Gomes N. C. M. Assessment of variation in bacterial composition among microhabitats in a mangrove environment using DGGE fingerprints and barcoded pyrosequencing. Plos One. 7, e29380 (2012). 22247774 10.1371/journal.pone.0029380 PMC3256149

[b27] Huang X. *et al.* Cel8H, a novel endoglucanase from the halophilic bacterium *Halomonas* sp. S66-4: Molecular cloning, heterogonous expression, and biochemical characterization. J. Microb. 48, 318–324 (2010). 10.1007/s12275-009-0188-5 20571949

[b28] Gao Z., Ruan L., Chen X., Zhang Y. & Xu X. A novel salt-tolerant endo-β-1,4-glucanase Cel5A in *Vibrio* sp. G21 isolated from mangrove soil. Appl. Microbiol. Biotechnol. 87, 1373–1382 (2010). 20393708 10.1007/s00253-010-2554-y

[b29] Femi-Ola T. O. & Babalola A. G. Microbiology of the gut of the kola nut weevil, Balanogastris kolae. J. Insect Sci. 12, 84 (2012). 23421598 10.1673/031.012.8401 PMC3596930

[b30] Kaltenpoth M. & Engl T. Defensive microbial symbionts in Hymenoptera. Funct Ecol. 28, 315–327 (2014).

[b31] Lam K., Thu K., Tsang M., Moore M. & Gries G. Bacteria on house fly eggs, *Musca domestica*, suppress fungal growth in chicken manure through nutrient depletion or antifungal metabolites. Naturwissenschaften. 96, 1127–1132 (2009). 19636523 10.1007/s00114-009-0574-1

[b32] Chung S. H. *et al.* Herbivore exploits orally secreted bacteria to suppress plant defences. PNAS. 110, 15728–15733 (2013). 24019469 10.1073/pnas.1308867110 PMC3785742

[b33] Senderovich Y. & Halpern M. Bacterial community composition associated with chironomid egg masses. J. Insect Sci. 12, 149 (2012). 23461272 10.1673/031.012.14901 PMC3646614

[b34] Scully E. D. *et al.* Metagenomic profiling reveals lignocellulose degrading system in a microbial community associated with a wood-feeding beetle. Plos One 8, e73827 (2013). 24023907 10.1371/journal.pone.0073827 PMC3762729

[b35] Wurl O. & Obbard J. P. A review of pollutants in the sea-surface microlayer (SML): a unique habitat for marine organisms. Mar. Pollut. Bull. 48, 1016–1030 (2004). 15172807 10.1016/j.marpolbul.2004.03.016

[b36] Azevedo J. S. N. *et al.* Spatial and temporal analysis of estuarine bacterioneuston and bacterioplankton using culture-dependent and culture-independent methodologies. Anton. Leeuw. 101, 819–835 (2012). 10.1007/s10482-012-9697-z 22258199

[b37] Han Z. M., Hong Y. D. & Zhao B. G. A study on pathogenicity of bacteria carried by pine wood nematodes. J. Phytop. 151, 683–689 (2003).

[b38] Proença D. N. *et al.* Diversity of bacteria associated with *Bursaphelenchus xylophilus* and other nematodes isolated from *Pinus pinaster* trees with pine wilt disease, Plos One. 5, e15191 (2010). 21151611 10.1371/journal.pone.0015191 PMC3000323

[b39] Vicente C. S. L., Nascimento F., Espada M., Mota M. & Oliveira S. Bacteria associated with the pinewood nematode *Bursaphelenchus xylophilus* collected in Portugal. Ant. Leeuw. 100, 477–481 (2011). 10.1007/s10482-011-9602-1 21656192

[b40] OEPP/EPPO. EPPO A1 and A2 lists of pests recommended for regulation as quarantine pests (2014).

[b41] Vicente C. S. L., Ikuyo Y., Mota M. & Hasegaw K. Pinewood nematode-associated bacteria contribute to oxidative stress resistance of *Bursaphelenchus xylophilus*. BMC Microb. 13, 299 (2013b). 10.1186/1471-2180-13-299 PMC3880045 24365493

[b42] Vicente C. S. L. *et al.* Characterization of bacteria associated with pinewood nematode *Bursaphelenchus xylophilus*. Plos One 7, e46661 (2012b). 23091599 10.1371/journal.pone.0046661 PMC3473040

[b43] Aksoy E. *et al.* Analysis of multiple tsetse fly populations in uganda reveals limited diversity and species-specific gut microbiota. Appl. Environ. Microbiol. 80, 4301–4312 (2014). 24814785 10.1128/AEM.00079-14 PMC4068677

[b44] Proença D. N., Fonseca L., Powers T. O., Abrantes I. M. O. & Morais P. V. Diversity of bacteria carried by pinewood nematode in USA and phylogenetic comparison with Isolates from other countries. Plos One. 9, e105190 (2014). 25127255 10.1371/journal.pone.0105190 PMC4134288

[b45] Peel M. C., Finlayson B. L. & McMahon T. A. Updated world map of the Köppen-Geiger climate classification, Hydrol. Earth Syst. Sci. 11, 1633–1644 (2007).

[b46] Geib S. M. *et al.* Effect of host tree species on cellulase activity and bacterial community composition in the gut of larval Asian longhorned beetle. Environ. Entomol. 38, 686–699 (2009). 19508777 10.1603/022.038.0320

[b47] Xiang Y., Wu X.-Q. & Zhou A.-D. Bacterial diversity and community structure in the pine wood nematode *Bursaphelenchus xylophilus* and *B. mucronatus* with different virulence by high-throughput sequencing of the 16S rDNA. Plos One. 10, e0137386 (2015). 26372013 10.1371/journal.pone.0137386 PMC4570786

[b48] ICNF. Informação geográfica – ICNF. Available at: http://www.icnf.pt/portal/florestas/prag-doe/ag-bn/nmp/infgeo (Accessed: 21th September 2015).

[b49] Mallez S. *et al.* Worldwide invasion routes of the pinewood nematode: What can we infer from population genetics analyses? Biol. Invasions. 17, 1199–1213 (2014).

[b50] Simon C. *et al.* Evolution, weighting, and phylogenetic utility of mitochondrial gene sequences and a compilation of conserved polymerase chain reaction primers. Ann. Entomol. Soc. Am. 87, 651–702 (1994).

[b51] PM 7/4 (3) Bursaphelenchus xylophilus. EPPO Bulletin 43, 105–118 (2013).

[b52] Cardoso J. M. S., Fonseca L. & Abrantes I. Direct molecular detection of the pinewood nematode, *Bursaphelenchus xylophilus*, from pine wood, bark and insect vector Eur. J. Plant. Pathol. 133, 419–425 (2012).

[b53] Lane D. J. *et al.* Rapid determination of 16S ribosomal RNA sequences for phylogenetic analyses. Proc. Natl. Acad. Sci. USA 82, 6955–6959 (1985). 2413450 10.1073/pnas.82.20.6955 PMC391288

[b54] Wang Y. & Qian P.-Y. Conservative fragments in bacterial 16S rRNA genes and primer design for 16S ribosomal DNA amplicons in metagenomic studies. Plos One. 4, e7401 (2009). 19816594 10.1371/journal.pone.0007401 PMC2754607

[b55] Edgar R. C. UPARSE: highly accurate OTU sequences from microbial amplicon reads. Nat. Methods 10, 996–998 (2013). 23955772 10.1038/nmeth.2604

[b56] Caporaso J. G. *et al.* QIIME allows analysis of high-throughput community sequencing data. Nat. Methods 7, 335–336 (2010). 20383131 10.1038/nmeth.f.303 PMC3156573

[b57] Shannon C. E. & Weaver W. *The mathematical theory of communication*. Urbana, IL: University of Illinois Press (1963).

[b58] Pielou E. C. Ecological diversity. New York: Wiley (1975).

[b59] Anderson M. J., Gorley R. N. & Clarke K. R. *PERMANOVA*+ *for PRIMER: Guide to Software and Statistical Methods*. PRIMER-E, Plymouth, UK (2008).

[b60] Fromin N. *et al.* Statistical analysis of denaturing gel electrophoresis (DGE) fingerprinting patterns. Environ. Microbiol. 4, 634–643 (2002). 12460271 10.1046/j.1462-2920.2002.00358.x

