# Supplementary material for: Bacterial community associated to the pine wilt disease insect vectors Monochamus galloprovincialis and Monochamus alternatus
Source: Sci Rep. 2016 Apr 5;6:23908. doi: 10.1038/srep23908 (PMC4820700; doi:10.1038/srep23908)
Supplement: Supplementary Information [file srep23908-s1.doc]

**Bacterial community associated to the pine wilt disease insect vectors *Monochamus galloprovincialis* and *Monochamus alternatus***

Marta Alves^1,2^, Anabela Pereira^1^, Patrícia Matos^1^, Joana Henriques^3^, Cláudia Vicente^4,5^, Takuya Aikawa^4^, Koichi Hasegawa^5^, Francisco Nascimento^3^, Manuel Mota^3^, António Correia^1*^, Isabel Henriques^2^

**Supplementary Information**

**Supplementary Table S1** – List of OTUs obtained and the respective taxonomy assignment using uclust default parameters. To each OTUs there are the corresponding values for the fraction of hits that correspond to that taxonomy and the number of uclust hits that were found (default: 3 is the maximum allowed).

| **OTUs** | **Domain** | **Phylum** | **Class** | **Order** | **Family** | **Genus** | **Species** | **Fraction** | **Hits found** |
| --- | --- | --- | --- | --- | --- | --- | --- | --- | --- |
| **OTU_1** | Bacteria | Proteobacteria | Gammaproteobacteria | Enterobacteriales | Enterobacteriaceae |  |  | 0.67 | 3 |
| **OTU_2** | Bacteria | Proteobacteria | Gammaproteobacteria | Enterobacteriales | Enterobacteriaceae |  |  | 0.67 | 3 |
| **OTU_3** | Bacteria | Proteobacteria | Gammaproteobacteria | Pseudomonadales | Pseudomonadaceae | *Pseudomonas* |  | 0.67 | 3 |
| **OTU_4** | Bacteria | Proteobacteria | Gammaproteobacteria | Oceanospirillales | Halomonadaceae | *Halomonas* |  | 0.67 | 3 |
| **OTU_5** | Bacteria | Proteobacteria | Gammaproteobacteria | Vibrionales | Vibrionaceae | *Vibrio* |  | 0.67 | 3 |
| **OTU_6** | Bacteria | Proteobacteria | Gammaproteobacteria | Enterobacteriales | Enterobacteriaceae |  |  | 1.00 | 3 |
| **OTU_7** | Bacteria | Proteobacteria | Gammaproteobacteria | Enterobacteriales | Enterobacteriaceae |  |  | 0.67 | 3 |
| **OTU_8** | Bacteria | Firmicutes | Bacilli | Lactobacillales | Leuconostocaceae | *Weissella* |  | 0.67 | 3 |
| **OTU_9** | Bacteria | Proteobacteria | Alphaproteobacteria | Rhodobacterales | Rhodobacteraceae | *Oceanicola* |  | 0.67 | 3 |
| **OTU_10** | Bacteria | Proteobacteria | Gammaproteobacteria | Xanthomonadales | Xanthomonadaceae | *Stenotrophomonas* |  | 0.67 | 3 |
| **OTU_11** | Bacteria | Proteobacteria | Betaproteobacteria | Burkholderiales | Comamonadaceae | *Comamonas* |  | 0.67 | 3 |
| **OTU_12** | Bacteria | Proteobacteria | Gammaproteobacteria | Enterobacteriales | Enterobacteriaceae |  |  | 1.00 | 3 |
| **OTU_13** | Bacteria | Proteobacteria | Gammaproteobacteria | Pseudomonadales | Pseudomonadaceae |  |  | 0.67 | 3 |
| **OTU_14** | Bacteria | Proteobacteria | Alphaproteobacteria | Rhodospirillales | Acetobacteraceae |  |  | 1.00 | 3 |
| **OTU_15** | Bacteria | Proteobacteria | Alphaproteobacteria | Rhodobacterales | Rhodobacteraceae | *Paracoccus* |  | 0.67 | 3 |
| **OTU_16** | Bacteria | Acidobacteria | Acidobacteriia | Acidobacteriales | Acidobacteriaceae |  |  | 0.67 | 3 |
| **OTU_17** | Bacteria | Bacteroidetes | Flavobacteriia | Flavobacteriales | [Weeksellaceae] | *Wautersiella* |  | 1.00 | 3 |
| **OTU_18** | Bacteria | Proteobacteria | Gammaproteobacteria | Pseudomonadales | Moraxellaceae | *Acinetobacter* |  | 0.67 | 3 |
| **OTU_19** | Bacteria | Proteobacteria | Alphaproteobacteria | Rhizobiales | Rhizobiaceae | *Rhizobium* |  | 0.67 | 3 |
| **OTU_20** | Bacteria | Firmicutes | Bacilli | Lactobacillales | Aerococcaceae |  |  | 1.00 | 3 |
| **OTU_21** | Bacteria | Bacteroidetes | Flavobacteriia | Flavobacteriales | [Weeksellaceae] | *Chryseobacterium* |  | 1.00 | 3 |
| **OTU_22** | Bacteria | Proteobacteria | Alphaproteobacteria | Caulobacterales | Caulobacteraceae |  |  | 1.00 | 3 |
| **OTU_23** | Bacteria | Proteobacteria | Gammaproteobacteria | Xanthomonadales | Xanthomonadaceae | *Luteibacter* | *rhizovicinus* | 0.67 | 3 |
| **OTU_24** | Bacteria | Bacteroidetes | [Saprospirae] | [Saprospirales] | Chitinophagaceae |  |  | 1.00 | 3 |
| **OTU_25** | Bacteria | Proteobacteria | Alphaproteobacteria | Sphingomonadales | Sphingomonadaceae | *Sphingomonas* | *wittichii* | 1.00 | 3 |
| **OTU_26** | Bacteria | Proteobacteria | Alphaproteobacteria | Sphingomonadales | Sphingomonadaceae |  |  | 1.00 | 3 |
| **OTU_27** | Bacteria | Acidobacteria | Acidobacteriia | Acidobacteriales | Acidobacteriaceae |  |  | 1.00 | 3 |
| **OTU_28** | Bacteria | Proteobacteria | Gammaproteobacteria | Vibrionales | Vibrionaceae | *Vibrio* |  | 0.67 | 3 |
| **OTU_29** | Bacteria | Firmicutes | Bacilli | Lactobacillales | Streptococcaceae | *Streptococcus* |  | 1.00 | 3 |
| **OTU_30** | Bacteria | Proteobacteria | Gammaproteobacteria | Pseudomonadales | Moraxellaceae | *Acinetobacter* |  | 1.00 | 3 |
| **OTU_31** | Bacteria | Proteobacteria | Alphaproteobacteria | Sphingomonadales | Sphingomonadaceae | *Sphingomonas* |  | 0.67 | 3 |
| **OTU_32** | Bacteria | Proteobacteria | Gammaproteobacteria | Xanthomonadales | Xanthomonadaceae |  |  | 0.67 | 3 |
| **OTU_33** | Bacteria | Proteobacteria | Gammaproteobacteria | Pseudomonadales | Pseudomonadaceae | *Pseudomonas* | *viridiflava* | 0.67 | 3 |
| **OTU_34** | Bacteria | Proteobacteria | Alphaproteobacteria | Sphingomonadales | Sphingomonadaceae |  |  | 0.67 | 3 |
| **OTU_35** | Bacteria | Acidobacteria | Solibacteres | Solibacterales | Solibacteraceae |  |  | 1.00 | 3 |
| **OTU_36** | Bacteria | Proteobacteria | Gammaproteobacteria | Pseudomonadales | Pseudomonadaceae | *Pseudomonas* |  | 1.00 | 3 |
| **OTU_37** | Bacteria | Proteobacteria | Betaproteobacteria | Burkholderiales | Comamonadaceae |  |  | 1.00 | 3 |
| **OTU_38** | Bacteria | Acidobacteria | Acidobacteriia | Acidobacteriales | Acidobacteriaceae |  |  | 0.67 | 3 |
| **OTU_39** | Bacteria | Proteobacteria | Gammaproteobacteria | Vibrionales | Pseudoalteromonadaceae | *Pseudoalteromonas* |  | 1.00 | 3 |
| **OTU_40** | Bacteria | Firmicutes | Bacilli | Bacillales | Staphylococcaceae | *Staphylococcus* | *epidermidis* | 1.00 | 3 |
| **OTU_41** | Bacteria | Proteobacteria | Alphaproteobacteria | Sphingomonadales | Sphingomonadaceae |  |  | 1.00 | 3 |
| **OTU_42** | Bacteria | Bacteroidetes | Sphingobacteriia | Sphingobacteriales | Sphingobacteriaceae |  |  | 1.00 | 3 |
| **OTU_43** | Bacteria | Proteobacteria | Gammaproteobacteria | Pseudomonadales | Moraxellaceae | *Acinetobacter* | *rhizosphaerae* | 0.67 | 3 |
| **OTU_44** | Bacteria | Acidobacteria | Acidobacteriia | Acidobacteriales | Acidobacteriaceae |  |  | 0.67 | 3 |
| **OTU_45** | Bacteria | Firmicutes | Bacilli | Bacillales | Bacillaceae | *Bacillus* | *cereus* | 0.67 | 3 |
| **OTU_46** | Bacteria | Proteobacteria | Alphaproteobacteria | Rhizobiales | Xanthobacteraceae | *Labrys* |  | 1.00 | 3 |
| **OTU_47** | Bacteria | Armatimonadetes | Armatimonadia | FW68 |  |  |  | 1.00 | 3 |
| **OTU_48** | Bacteria | Proteobacteria | Alphaproteobacteria | Rhizobiales | Bradyrhizobiaceae | *Bradyrhizobium* |  | 0.67 | 3 |
| **OTU_49** | Bacteria | Bacteroidetes | Sphingobacteriia | Sphingobacteriales | Sphingobacteriaceae |  |  | 1.00 | 3 |
| **OTU_50** | Bacteria | Acidobacteria | Acidobacteriia | Acidobacteriales | Acidobacteriaceae | *Terriglobus* |  | 1.00 | 3 |
| **OTU_51** | Bacteria | Firmicutes | Clostridia | Clostridiales | [Mogibacteriaceae] |  |  | 1.00 | 3 |
| **OTU_53** | Bacteria | Bacteroidetes | Sphingobacteriia | Sphingobacteriales | Sphingobacteriaceae |  |  | 1.00 | 3 |
| **OTU_54** | Bacteria | Proteobacteria | Alphaproteobacteria | Rhizobiales | Methylocystaceae |  |  | 1.00 | 3 |
| **OTU_55** | Unassigned |  |  |  |  |  |  | 1.00 | 1 |
| **OTU_56** | Bacteria | Proteobacteria | Gammaproteobacteria | Pasteurellales | Pasteurellaceae | *Haemophilus* | *parainfluenzae* | 0.67 | 3 |
| **OTU_57** | Bacteria | Acidobacteria | Acidobacteriia | Acidobacteriales | Acidobacteriaceae |  |  | 1.00 | 3 |
| **OTU_58** | Bacteria | Proteobacteria | Gammaproteobacteria | Alteromonadales | Shewanellaceae | *Shewanella* |  | 1.00 | 3 |
| **OTU_59** | Bacteria | Proteobacteria | Alphaproteobacteria | Rhizobiales | Rhizobiaceae | *Agrobacterium* |  | 0.67 | 3 |
| **OTU_60** | Bacteria | Cyanobacteria | Nostocophycideae | Nostocales | Nostocaceae |  |  | 0.67 | 3 |
| **OTU_61** | Bacteria | Acidobacteria | Acidobacteriia | Acidobacteriales | Acidobacteriaceae |  |  | 0.67 | 3 |
| **OTU_62** | Bacteria | Proteobacteria | Betaproteobacteria | Burkholderiales | Burkholderiaceae | *Burkholderia* | *andropogonis* | 0.67 | 3 |
| **OTU_63** | Bacteria | Acidobacteria | [Chloracidobacteria] | RB41 | Ellin6075 |  |  | 1.00 | 3 |
| **OTU_64** | Bacteria | Proteobacteria | Alphaproteobacteria | Rhizobiales |  |  |  | 0.67 | 3 |
| **OTU_65** | Bacteria | Bacteroidetes | Sphingobacteriia | Sphingobacteriales | Sphingobacteriaceae |  |  | 1.00 | 3 |
| **OTU_66** | Bacteria | Bacteroidetes | Sphingobacteriia | Sphingobacteriales | Sphingobacteriaceae |  |  | 1.00 | 3 |
| **OTU_67** | Bacteria | Bacteroidetes | Sphingobacteriia | Sphingobacteriales | Sphingobacteriaceae |  |  | 1.00 | 3 |
| **OTU_68** | Bacteria | Proteobacteria | Betaproteobacteria | Burkholderiales | Comamonadaceae |  |  | 1.00 | 3 |
| **OTU_69** | Bacteria | Proteobacteria | Gammaproteobacteria | Enterobacteriales | Enterobacteriaceae | *Enterobacter* |  | 0.67 | 3 |
| **OTU_70** | Bacteria | Bacteroidetes | Sphingobacteriia | Sphingobacteriales | Sphingobacteriaceae |  |  | 1.00 | 3 |
| **OTU_71** | Bacteria | Proteobacteria | Alphaproteobacteria | Rhizobiales | Rhizobiaceae |  |  | 0.67 | 3 |
| **OTU_72** | Bacteria | Proteobacteria | Gammaproteobacteria | Pseudomonadales | Moraxellaceae | *Enhydrobacter* |  | 1.00 | 3 |
| **OTU_73** | Bacteria | Acidobacteria | Acidobacteriia | Acidobacteriales | Acidobacteriaceae |  |  | 1.00 | 3 |
| **OTU_74** | Unassigned |  |  |  |  |  |  | 1.00 | 1 |
| **OTU_75** | Bacteria | Actinobacteria | Actinobacteria | Actinomycetales | Microbacteriaceae | *Salinibacterium* |  | 0.67 | 3 |
| **OTU_76** | Bacteria | Proteobacteria | Gammaproteobacteria | Xanthomonadales | Xanthomonadaceae |  |  | 1.00 | 3 |
| **OTU_77** | Bacteria | Bacteroidetes | [Saprospirae] | [Saprospirales] | Chitinophagaceae |  |  | 1.00 | 3 |
| **OTU_78** | Bacteria | Proteobacteria | Betaproteobacteria | Neisseriales | Neisseriaceae | *Neisseria* |  | 1.00 | 3 |
| **OTU_79** | Bacteria | Proteobacteria | Alphaproteobacteria | Rhodospirillales | Acetobacteraceae | *Acidisoma* |  | 0.67 | 3 |
| **OTU_80** | Bacteria | TM7 | TM7-1 |  |  |  |  | 1.00 | 3 |
| **OTU_82** | Bacteria | Proteobacteria | Alphaproteobacteria | Rhizobiales | Beijerinckiaceae |  |  | 0.67 | 3 |
| **OTU_83** | Bacteria | Proteobacteria | Betaproteobacteria | Burkholderiales | Comamonadaceae | *Comamonas* |  | 0.67 | 3 |
| **OTU_84** | Bacteria | Bacteroidetes | Cytophagia | Cytophagales |  |  |  | 1.00 | 3 |
| **OTU_85** | Bacteria | Proteobacteria | Betaproteobacteria | Burkholderiales | Comamonadaceae | *Delftia* |  | 1.00 | 3 |
| **OTU_87** | Bacteria | Proteobacteria | Alphaproteobacteria | Caulobacterales | Caulobacteraceae |  |  | 1.00 | 3 |
| **OTU_88** | Bacteria | Proteobacteria | Alphaproteobacteria | Rhizobiales | Methylocystaceae |  |  | 1.00 | 3 |
| **OTU_89** | Bacteria | Proteobacteria | Alphaproteobacteria | Rhizobiales | Methylocystaceae |  |  | 1.00 | 3 |
| **OTU_90** | Bacteria | Proteobacteria | Gammaproteobacteria | Oceanospirillales | Halomonadaceae | *Halomonas* |  | 1.00 | 3 |
| **OTU_91** | Bacteria | Bacteroidetes | Sphingobacteriia | Sphingobacteriales | Sphingobacteriaceae |  |  | 1.00 | 3 |
| **OTU_92** | Bacteria | Proteobacteria | Alphaproteobacteria | Rhizobiales | Rhizobiaceae | *Kaistia* |  | 1.00 | 3 |
| **OTU_93** | Bacteria | Proteobacteria | Gammaproteobacteria | Xanthomonadales | Xanthomonadaceae |  |  | 0.67 | 3 |
| **OTU_94** | Bacteria | Bacteroidetes | [Saprospirae] | [Saprospirales] | Chitinophagaceae |  |  | 1.00 | 3 |
| **OTU_95** | Bacteria | Actinobacteria | Acidimicrobiia | Acidimicrobiales |  |  |  | 1.00 | 3 |
| **OTU_96** | Bacteria | Proteobacteria | Deltaproteobacteria | Myxococcales |  |  |  | 1.00 | 3 |
| **OTU_97** | Bacteria | Proteobacteria | Deltaproteobacteria | Myxococcales | Cystobacterineae |  |  | 1.00 | 3 |
| **OTU_98** | Bacteria | Proteobacteria | Gammaproteobacteria | Enterobacteriales | Enterobacteriaceae |  |  | 1.00 | 3 |
| **OTU_99** | Bacteria | Proteobacteria | Betaproteobacteria | Burkholderiales | Burkholderiaceae | *Burkholderia* |  | 0.67 | 3 |
| **OTU_100** | Bacteria | Proteobacteria | Alphaproteobacteria | Rhodospirillales | Acetobacteraceae |  |  | 1.00 | 3 |
| **OTU_101** | Bacteria | Cyanobacteria | Synechococcophycideae | Synechococcales | Synechococcaceae | *Synechococcus* |  | 1.00 | 3 |
| **OTU_102** | Bacteria | Armatimonadetes | Armatimonadia | FW68 |  |  |  | 1.00 | 3 |
| **OTU_103** | Bacteria | Proteobacteria | Betaproteobacteria | Burkholderiales | Comamonadaceae |  |  | 0.67 | 3 |
| **OTU_104** | Bacteria | Acidobacteria | Acidobacteriia | Acidobacteriales | Acidobacteriaceae |  |  | 0.67 | 3 |
| **OTU_105** | Bacteria | Proteobacteria | Alphaproteobacteria | Rhizobiales | Methylobacteriaceae | *Methylobacterium* |  | 0.67 | 3 |
| **OTU_106** | Bacteria | Proteobacteria | Alphaproteobacteria | Rhodospirillales | Acetobacteraceae |  |  | 1.00 | 3 |
| **OTU_107** | Bacteria | Proteobacteria | Alphaproteobacteria | Sphingomonadales | Sphingomonadaceae | *Sphingomonas* | *wittichii* | 1.00 | 3 |
| **OTU_108** | Bacteria | Proteobacteria | Alphaproteobacteria | Rickettsiales | Pelagibacteraceae |  |  | 1.00 | 3 |
| **OTU_109** | Bacteria | Proteobacteria | Gammaproteobacteria | Oceanospirillales | Halomonadaceae | *Chromohalobacter* |  | 0.67 | 3 |
| **OTU_110** | Bacteria | Proteobacteria | Betaproteobacteria | Burkholderiales | Burkholderiaceae | *Burkholderia* |  | 0.67 | 3 |
| **OTU_111** | Bacteria | Proteobacteria | Gammaproteobacteria | Oceanospirillales | Halomonadaceae | *Candidatus Portiera* |  | 1.00 | 3 |
| **OTU_112** | Bacteria | Proteobacteria | Alphaproteobacteria | Rhodobacterales | Rhodobacteraceae |  |  | 1.00 | 3 |
| **OTU_113** | Bacteria | Proteobacteria | Alphaproteobacteria | Sphingomonadales | Sphingomonadaceae | *Sphingomonas* |  | 1.00 | 3 |
| **OTU_114** | Bacteria | Bacteroidetes | Flavobacteriia | Flavobacteriales | Flavobacteriaceae |  |  | 1.00 | 3 |
| **OTU_115** | Bacteria | Proteobacteria | Alphaproteobacteria | Sphingomonadales | Sphingomonadaceae |  |  | 0.67 | 3 |
| **OTU_117** | Bacteria | Actinobacteria | Actinobacteria | Actinomycetales | Mycobacteriaceae | *Mycobacterium* |  | 1.00 | 3 |
| **OTU_119** | Bacteria | Proteobacteria | Alphaproteobacteria | Rhodospirillales | Acetobacteraceae |  |  | 1.00 | 3 |
| **OTU_120** | Bacteria | Proteobacteria | Betaproteobacteria | Burkholderiales | Oxalobacteraceae |  |  | 0.67 | 3 |
| **OTU_121** | Bacteria | Bacteroidetes | Sphingobacteriia | Sphingobacteriales | Sphingobacteriaceae | *Pedobacter* |  | 1.00 | 3 |
| **OTU_122** | Bacteria | Proteobacteria | Alphaproteobacteria | Rhodospirillales | Acetobacteraceae |  |  | 1.00 | 3 |
| **OTU_123** | Bacteria | Bacteroidetes | Sphingobacteriia | Sphingobacteriales | Sphingobacteriaceae | *Pedobacter* |  | 1.00 | 3 |
| **OTU_124** | Bacteria | Firmicutes | Bacilli | Lactobacillales | Streptococcaceae | *Lactococcus* |  | 0.67 | 3 |
| **OTU_125** | Bacteria | TM7 | TM7-1 |  |  |  |  | 1.00 | 3 |
| **OTU_126** | Bacteria | Proteobacteria | Gammaproteobacteria | Legionellales |  |  |  | 1.00 | 3 |
| **OTU_127** | Bacteria | Proteobacteria | Gammaproteobacteria | Xanthomonadales | Xanthomonadaceae |  |  | 0.67 | 3 |
| **OTU_128** | Bacteria | Proteobacteria | Alphaproteobacteria | Rhizobiales | Methylobacteriaceae | *Methylobacterium* |  | 1.00 | 3 |
| **OTU_129** | Bacteria | Proteobacteria | Alphaproteobacteria | Rhizobiales | Phyllobacteriaceae | *Phyllobacterium* |  | 1.00 | 3 |
| **OTU_131** | Bacteria | Proteobacteria | Alphaproteobacteria | Rhizobiales | Beijerinckiaceae |  |  | 0.67 | 3 |
| **OTU_132** | Bacteria | Proteobacteria | Alphaproteobacteria | Rhodospirillales | Acetobacteraceae | *Roseococcus* |  | 1.00 | 3 |
| **OTU_133** | Bacteria | Proteobacteria | Betaproteobacteria | Burkholderiales | Comamonadaceae | *Methylibium* |  | 0.67 | 3 |
| **OTU_134** | Bacteria | Proteobacteria | Alphaproteobacteria | Caulobacterales | Caulobacteraceae |  |  | 1.00 | 3 |
| **OTU_135** | Bacteria | Proteobacteria | Gammaproteobacteria | Oceanospirillales | Halomonadaceae | *Candidatus Portiera* |  | 1.00 | 3 |
| **OTU_136** | Bacteria | Proteobacteria | Deltaproteobacteria | Myxococcales |  |  |  | 1.00 | 3 |
| **OTU_137** | Bacteria | Actinobacteria | Acidimicrobiia | Acidimicrobiales |  |  |  | 1.00 | 3 |
| **OTU_138** | Bacteria | Proteobacteria | Alphaproteobacteria | Rhodospirillales | Acetobacteraceae |  |  | 0.67 | 3 |
| **OTU_139** | Bacteria | TM7 | TM7-3 |  |  |  |  | 1.00 | 3 |
| **OTU_140** | Bacteria | Proteobacteria | Gammaproteobacteria | Enterobacteriales | Enterobacteriaceae |  |  | 0.67 | 3 |
| **OTU_141** | Bacteria | Proteobacteria | Betaproteobacteria | Burkholderiales | Comamonadaceae |  |  | 1.00 | 3 |
| **OTU_143** | Bacteria | Proteobacteria | Alphaproteobacteria |  |  |  |  | 1.00 | 3 |
| **OTU_144** | Bacteria | Cyanobacteria | Synechococcophycideae | Synechococcales | Synechococcaceae | *Prochlorococcus* |  | 1.00 | 3 |
| **OTU_145** | Bacteria | Proteobacteria | Alphaproteobacteria | Rickettsiales | Pelagibacteraceae |  |  | 1.00 | 3 |
| **OTU_146** | Bacteria | Proteobacteria | Gammaproteobacteria | Pseudomonadales | Moraxellaceae |  |  | 1.00 | 3 |
| **OTU_147** | Bacteria | Proteobacteria | Alphaproteobacteria | Sphingomonadales | Sphingomonadaceae | *Sphingomonas* | *wittichii* | 1.00 | 3 |
| **OTU_149** | Bacteria | Proteobacteria | Gammaproteobacteria | Pseudomonadales | Moraxellaceae | *Acinetobacter* |  | 1.00 | 3 |
| **OTU_150** | Bacteria | Acidobacteria | Acidobacteriia | Acidobacteriales | Acidobacteriaceae |  |  | 1.00 | 3 |
| **OTU_151** | Bacteria | Cyanobacteria |  |  |  |  |  | 1.00 | 1 |
| **OTU_152** | Bacteria | Bacteroidetes | [Saprospirae] | [Saprospirales] | Chitinophagaceae |  |  | 0.67 | 3 |
| **OTU_153** | Bacteria | Proteobacteria | Alphaproteobacteria |  |  |  |  | 1.00 | 3 |
| **OTU_154** | Bacteria | Actinobacteria | Actinobacteria | Actinomycetales | Nocardioidaceae |  |  | 1.00 | 3 |
| **OTU_155** | Unassigned |  |  |  |  |  |  | 1.00 | 1 |
| **OTU_156** | Bacteria | SAR406 | AB16 | Arctic96B-7 | A714017 | *SGSH944* |  | 1.00 | 3 |
| **OTU_157** | Bacteria | Bacteroidetes | Flavobacteriia | Flavobacteriales | Flavobacteriaceae | *Sediminicola* |  | 1.00 | 3 |
| **OTU_159** | Bacteria | Armatimonadetes | Armatimonadia | FW68 |  |  |  | 1.00 | 3 |
| **OTU_160** | Bacteria | Bacteroidetes | Cytophagia | Cytophagales | Cytophagaceae | *Hymenobacter* |  | 1.00 | 3 |
| **OTU_161** | Bacteria | Proteobacteria | Alphaproteobacteria | Rhodospirillales | Acetobacteraceae |  |  | 1.00 | 3 |
| **OTU_162** | Bacteria | Bacteroidetes | [Saprospirae] | [Saprospirales] | Chitinophagaceae |  |  | 1.00 | 3 |
| **OTU_164** | Bacteria | Acidobacteria | Acidobacteriia | Acidobacteriales | Acidobacteriaceae |  |  | 1.00 | 3 |
| **OTU_165** | Bacteria | Acidobacteria | Acidobacteriia | Acidobacteriales | Acidobacteriaceae |  |  | 1.00 | 3 |
| **OTU_166** | Bacteria | TM7 | TM7-1 |  |  |  |  | 1.00 | 3 |
| **OTU_167** | Bacteria | Proteobacteria | Betaproteobacteria | Burkholderiales | Oxalobacteraceae |  |  | 0.67 | 3 |
| **OTU_168** | Bacteria | Proteobacteria | Deltaproteobacteria | Myxococcales |  |  |  | 1.00 | 3 |
| **OTU_169** | Bacteria | Proteobacteria | Gammaproteobacteria | Pseudomonadales | Pseudomonadaceae | *Pseudomonas* |  | 1.00 | 3 |
| **OTU_170** | Bacteria | SAR406 | AB16 | ZA3648c | AEGEAN_185 |  |  | 1.00 | 3 |
| **OTU_171** | Bacteria | Proteobacteria | Gammaproteobacteria | Enterobacteriales | Enterobacteriaceae |  |  | 0.67 | 3 |
| **OTU_172** | Bacteria | Armatimonadetes | Armatimonadia | FW68 |  |  |  | 1.00 | 3 |
| **OTU_173** | Bacteria | Proteobacteria | Gammaproteobacteria | Enterobacteriales | Enterobacteriaceae |  |  | 0.67 | 3 |
| **OTU_174** | Bacteria | Proteobacteria | Gammaproteobacteria | Oceanospirillales | Halomonadaceae | *Candidatus Portiera* |  | 1.00 | 3 |
| **OTU_175** | Unassigned |  |  |  |  |  |  | 1.00 | 1 |
| **OTU_176** | Bacteria | Proteobacteria | Gammaproteobacteria | Alteromonadales | Alteromonadaceae | *Marinobacter* |  | 1.00 | 3 |
| **OTU_177** | Bacteria | Proteobacteria | Betaproteobacteria | Burkholderiales | Burkholderiaceae | *Burkholderia* | *andropogonis* | 1.00 | 3 |
| **OTU_178** | Bacteria | Bacteroidetes | Cytophagia | Cytophagales | Cytophagaceae | *Hymenobacter* |  | 1.00 | 3 |
| **OTU_179** | Bacteria | Proteobacteria | Alphaproteobacteria | Rhizobiales | Hyphomicrobiaceae | *Devosia* |  | 1.00 | 3 |
| **OTU_180** | Bacteria | Proteobacteria | Alphaproteobacteria | Rhizobiales | Methylocystaceae |  |  | 1.00 | 3 |
| **OTU_181** | Bacteria | Proteobacteria | Alphaproteobacteria | Rhizobiales |  |  |  | 1.00 | 3 |
| **OTU_182** | Bacteria | Proteobacteria | Alphaproteobacteria | Sphingomonadales | Sphingomonadaceae | *Sphingomonas* | *wittichii* | 1.00 | 3 |
| **OTU_183** | Bacteria | Proteobacteria | Alphaproteobacteria | Rickettsiales | Pelagibacteraceae |  |  | 1.00 | 3 |
| **OTU_185** | Unassigned |  |  |  |  |  |  | 1.00 | 1 |
| **OTU_186** | Bacteria | Proteobacteria | Alphaproteobacteria | Rhodobacterales | Rhodobacteraceae |  |  | 0.67 | 3 |
| **OTU_187** | Bacteria | Chloroflexi | Ktedonobacteria | Elev-1554 |  |  |  | 1.00 | 2 |
| **OTU_188** | Bacteria | Proteobacteria | Gammaproteobacteria | Aeromonadales | Aeromonadaceae |  |  | 0.67 | 3 |
| **OTU_189** | Bacteria | Acidobacteria | Solibacteres | Solibacterales |  |  |  | 0.67 | 3 |
| **OTU_190** | Bacteria | Bacteroidetes | Flavobacteriia | Flavobacteriales | [Weeksellaceae] | *Chryseobacterium* |  | 1.00 | 3 |
| **OTU_191** | Bacteria | Actinobacteria | Thermoleophilia | Solirubrobacterales | Patulibacteraceae | *Patulibacter* |  | 1.00 | 3 |
| **OTU_192** | Bacteria | Proteobacteria | Gammaproteobacteria | Pseudomonadales | Pseudomonadaceae | *Pseudomonas* |  | 0.67 | 3 |
| **OTU_193** | Bacteria | Chloroflexi | SAR202 |  |  |  |  | 1.00 | 3 |
| **OTU_194** | Bacteria | Proteobacteria | Betaproteobacteria | Burkholderiales | Burkholderiaceae |  |  | 1.00 | 3 |
| **OTU_195** | Bacteria | SAR406 | AB16 | Arctic96B-7 | A714017 | *ZA3312c* |  | 1.00 | 3 |
| **OTU_196** | Bacteria | Proteobacteria | Alphaproteobacteria |  |  |  |  | 1.00 | 3 |
| **OTU_197** | Bacteria | Actinobacteria | Actinobacteria | Actinomycetales | Streptomycetaceae | *Streptomyces* |  | 1.00 | 3 |
| **OTU_198** | Bacteria | Proteobacteria | Alphaproteobacteria |  |  |  |  | 1.00 | 3 |
| **OTU_199** | Bacteria | Actinobacteria | Actinobacteria | Actinomycetales | Nocardiaceae | *Rhodococcus* |  | 0.67 | 3 |
| **OTU_200** | Bacteria | Proteobacteria | Gammaproteobacteria | Enterobacteriales | Enterobacteriaceae |  |  | 1.00 | 3 |
| **OTU_201** | Bacteria | Bacteroidetes | Sphingobacteriia | Sphingobacteriales | Sphingobacteriaceae |  |  | 0.67 | 3 |
| **OTU_202** | Bacteria | Firmicutes | Bacilli | Bacillales | [Thermicanaceae] | *Thermicanus* |  | 1.00 | 2 |
| **OTU_203** | Bacteria | Proteobacteria | Alphaproteobacteria | Rhodospirillales | Acetobacteraceae |  |  | 0.67 | 3 |
| **OTU_204** | Bacteria | Chloroflexi | Ktedonobacteria | Thermogemmatisporales | Thermogemmatisporaceae |  |  | 1.00 | 3 |
| **OTU_205** | Bacteria | WPS-2 |  |  |  |  |  | 1.00 | 3 |
| **OTU_206** | Bacteria | Proteobacteria | Gammaproteobacteria | Enterobacteriales | Enterobacteriaceae | *Erwinia* |  | 0.67 | 3 |
| **OTU_207** | Bacteria | Proteobacteria | Gammaproteobacteria | Alteromonadales | Alteromonadaceae | *Alteromonas* |  | 1.00 | 3 |
| **OTU_208** | Bacteria | Proteobacteria | Gammaproteobacteria | Pseudomonadales | Pseudomonadaceae | *Pseudomonas* |  | 0.67 | 3 |
| **OTU_209** | Bacteria | Proteobacteria | Alphaproteobacteria | Rhizobiales | Beijerinckiaceae |  |  | 0.67 | 3 |
| **OTU_210** | Bacteria | Proteobacteria | Alphaproteobacteria | Sphingomonadales | Sphingomonadaceae |  |  | 1.00 | 3 |
| **OTU_211** | Bacteria | Firmicutes | Bacilli | Bacillales | Paenibacillaceae |  |  | 1.00 | 3 |
| **OTU_212** | Bacteria | Proteobacteria | Gammaproteobacteria | Pseudomonadales | Moraxellaceae | *Acinetobacter* |  | 1.00 | 3 |
| **OTU_213** | Bacteria | Acidobacteria | Acidobacteria-6 | iii1-15 |  |  |  | 1.00 | 3 |
| **OTU_214** | Bacteria | Proteobacteria | Alphaproteobacteria | Rhizobiales | Methylocystaceae |  |  | 1.00 | 3 |
| **OTU_215** | Bacteria | Proteobacteria | Deltaproteobacteria | Bdellovibrionales | Bdellovibrionaceae | *Bdellovibrio* |  | 1.00 | 1 |
| **OTU_216** | Bacteria | Acidobacteria | Solibacteres | Solibacterales | Solibacteraceae |  |  | 1.00 | 3 |
| **OTU_217** | Bacteria | Proteobacteria | Alphaproteobacteria | Caulobacterales | Caulobacteraceae |  |  | 1.00 | 3 |
| **OTU_218** | Bacteria | TM7 | TM7-1 |  |  |  |  | 1.00 | 3 |
| **OTU_219** | Bacteria | Proteobacteria | Betaproteobacteria | Burkholderiales | Burkholderiaceae | *Burkholderia* |  | 0.67 | 3 |
| **OTU_220** | Bacteria | Proteobacteria | Betaproteobacteria | Burkholderiales | Comamonadaceae |  |  | 0.67 | 3 |
| **OTU_221** | Bacteria | Actinobacteria | Actinobacteria | Actinomycetales | Dermacoccaceae | *Dermacoccus* |  | 1.00 | 3 |
| **OTU_222** | Bacteria | Firmicutes | Bacilli | Bacillales | Bacillaceae | *Bacillus* | *coagulans* | 0.67 | 3 |
| **OTU_223** | Bacteria | Proteobacteria | Gammaproteobacteria | Pseudomonadales | Moraxellaceae | *Psychrobacter* |  | 0.67 | 3 |
| **OTU_224** | Unassigned |  |  |  |  |  |  | 1.00 | 1 |
| **OTU_225** | Bacteria | Actinobacteria | Acidimicrobiia | Acidimicrobiales |  |  |  | 1.00 | 3 |
| **OTU_226** | Bacteria | Proteobacteria | Deltaproteobacteria |  |  |  |  | 1.00 | 3 |
| **OTU_227** | Bacteria | Bacteroidetes | Flavobacteriia | Flavobacteriales |  |  |  | 1.00 | 3 |

**Supplementary Table S2** – Relative abundance of each phylum in each insect sample: a) *Monochamus galloprovincialis* samples; b) *Monochamus alternatus* samples.

a)

| **Phylum/Samples** | ***M. galloprovincialis*** | | | | | | | | | | |
| --- | --- | --- | --- | --- | --- | --- | --- | --- | --- | --- | --- |
|  | **Comporta** | | | | | | **Mortágua** | | | | |
|  | MG.PC37 | MG.PC41 | MG.PC43 | MG.PC42 | MG.PC46 | MG.PC48 | MG.PM22 | MG.PM24 | MG.PM29 | MG.PM32 | MG.PM36 |
| **Proteobacteria** | 99,28 | 92,89 | 98,24 | 98,58 | 96,15 | 99,96 | 99,92 | 98,95 | 99,57 | 100,00 | 99,40 |
| **Firmicutes** | 0,72 | 3,70 | 0,00 | 0,00 | 2,85 | 0,00 | 0,00 | 0,00 | 0,03 | 0,00 | 0,46 |
| **Bacteroidetes** | 0,00 | 2,56 | 1,76 | 0,69 | 1,01 | 0,04 | 0,08 | 1,05 | 0,40 | 0,00 | 0,14 |
| **Acidobacteria** | 0,00 | 0,85 | 0,00 | 0,00 | 0,00 | 0,00 | 0,00 | 0,00 | 0,00 | 0,00 | 0,00 |
| **Actinobacteria** | 0,00 | 0,00 | 0,00 | 0,73 | 0,00 | 0,00 | 0,00 | 0,00 | 0,00 | 0,00 | 0,00 |
| **Cyanobacteria** | 0,00 | 0,00 | 0,00 | 0,00 | 0,00 | 0,00 | 0,00 | 0,00 | 0,00 | 0,00 | 0,00 |
| **SAR406** | 0,00 | 0,00 | 0,00 | 0,00 | 0,00 | 0,00 | 0,00 | 0,00 | 0,00 | 0,00 | 0,00 |
| **TM7** | 0,00 | 0,00 | 0,00 | 0,00 | 0,00 | 0,00 | 0,00 | 0,00 | 0,00 | 0,00 | 0,00 |
| **Chloroflexi** | 0,00 | 0,00 | 0,00 | 0,00 | 0,00 | 0,00 | 0,00 | 0,00 | 0,00 | 0,00 | 0,00 |
| **Armatimonadetes** | 0,00 | 0,00 | 0,00 | 0,00 | 0,00 | 0,00 | 0,00 | 0,00 | 0,00 | 0,00 | 0,00 |
| **WPS-2** | 0,00 | 0,00 | 0,00 | 0,00 | 0,00 | 0,00 | 0,00 | 0,00 | 0,00 | 0,00 | 0,00 |
| **Unassigned** | 0,00 | 0,00 | 0,00 | 0,00 | 0,00 | 0,00 | 0,00 | 0,00 | 0,00 | 0,00 | 0,00 |

b)

| **Phylum/Samples** | ***M. alternatus*** | | | | | |
| --- | --- | --- | --- | --- | --- | --- |
|  | **Hikobe** | | | | | |
|  | MA.JH55 | MA.JH56 | MA.JH59 | MA.JH60 | MA.JH61 | MA.JH66 |
| **Proteobacteria** | 95,78 | 93,24 | 72,69 | 56,04 | 99,85 | 94,79 |
| **Firmicutes** | 2,47 | 0,00 | 0,00 | 32,55 | 0,00 | 0,95 |
| **Bacteroidetes** | 0,04 | 5,84 | 8,23 | 1,25 | 0,02 | 2,24 |
| **Acidobacteria** | 0,91 | 0,02 | 18,15 | 6,20 | 0,12 | 1,37 |
| **Actinobacteria** | 0,13 | 0,07 | 0,75 | 0,07 | 0,01 | 0,06 |
| **Cyanobacteria** | 0,45 | 0,01 | 0,00 | 1,07 | 0,01 | 0,00 |
| **SAR406** | 0,15 | 0,00 | 0,00 | 0,07 | 0,01 | 0,00 |
| **TM7** | 0,00 | 0,00 | 0,19 | 0,02 | 0,00 | 0,49 |
| **Chloroflexi** | 0,04 | 0,00 | 0,00 | 0,06 | 0,00 | 0,00 |
| **Armatimonadetes** | 0,00 | 0,00 | 0,00 | 1,39 | 0,00 | 0,00 |
| **WPS-2** | 0,00 | 0,00 | 0,00 | 0,07 | 0,00 | 0,00 |
| **Unassigned** | 0,04 | 0,82 | 0,00 | 1,20 | 0,00 | 0,10 |

**Supplementary Table S3** – Relative abundance of each order in each insect sample, a) *Monochamus galloprovincialis* samples; b) *Monochamus alternatus* samples.

a)

| **Order/Samples** | ***M. galloprovincialis*** | | | | | | | | | | |
| --- | --- | --- | --- | --- | --- | --- | --- | --- | --- | --- | --- |
|  | **Comporta** | | | | | | **Mortágua** | | | | |
|  | **MG.PC37** | **MG.PC41** | **MG.PC43** | **MG.PC42** | **MG.PC46** | **MG.PC48** | **MG.PM22** | **MG.PM24** | **MG.PM29** | **MG.PM32** | **MG.PM36** |
| **[Saprospirales]** | 0,00 | 0,00 | 0,00 | 0,00 | 0,00 | 0,00 | 0,00 | 0,00 | 0,00 | 0,00 | 0,00 |
| **Acidobacteriales** | 0,00 | 0,00 | 0,00 | 0,00 | 0,00 | 0,00 | 0,00 | 0,00 | 0,00 | 0,00 | 0,00 |
| **Acidimicrobiales** | 0,00 | 0,00 | 0,00 | 0,73 | 0,00 | 0,00 | 0,00 | 0,00 | 0,00 | 0,00 | 0,00 |
| **Actinomycetales** | 0,00 | 0,00 | 0,00 | 0,00 | 0,00 | 0,00 | 0,00 | 0,00 | 0,00 | 0,00 | 0,00 |
| **Aeromonadales** | 0,00 | 0,00 | 0,00 | 0,00 | 0,00 | 0,00 | 0,00 | 0,00 | 0,00 | 0,00 | 0,00 |
| **Alteromonadales** | 0,00 | 1,21 | 0,00 | 0,00 | 0,00 | 0,00 | 0,00 | 0,00 | 0,00 | 0,00 | 0,00 |
| **Arctic96B-7** | 0,00 | 0,00 | 0,00 | 0,00 | 0,00 | 0,00 | 0,00 | 0,00 | 0,00 | 0,00 | 0,00 |
| **Bdellovibrionales** | 0,00 | 0,00 | 0,00 | 0,00 | 0,00 | 0,00 | 0,00 | 0,00 | 0,00 | 0,00 | 0,00 |
| **Bacillales** | 0,72 | 0,04 | 0,00 | 0,00 | 0,00 | 0,00 | 0,00 | 0,00 | 0,03 | 0,00 | 0,46 |
| **Burkholderiales** | 9,88 | 0,06 | 2,79 | 3,62 | 6,84 | 2,22 | 0,00 | 0,00 | 1,95 | 0,31 | 0,39 |
| **Clostridiales** | 0,00 | 0,00 | 0,00 | 0,00 | 0,00 | 0,00 | 0,00 | 0,00 | 0,00 | 0,00 | 0,00 |
| **Caulobacterales** | 0,00 | 0,00 | 0,00 | 0,00 | 1,52 | 0,00 | 0,00 | 0,00 | 0,00 | 0,00 | 0,00 |
| **Cytophagales** | 0,00 | 0,00 | 0,00 | 0,00 | 0,00 | 0,00 | 0,00 | 1,05 | 0,00 | 0,00 | 0,00 |
| **Enterobacteriales** | 10,76 | 13,97 | 26,59 | 6,32 | 18,48 | 82,71 | 10,72 | 8,77 | 54,74 | 76,82 | 74,16 |
| **Elev-1554** | 0,00 | 0,00 | 0,00 | 0,00 | 0,00 | 0,00 | 0,00 | 0,00 | 0,00 | 0,00 | 0,00 |
| **Flavobacteriales** | 0,00 | 2,56 | 1,76 | 0,69 | 1,01 | 0,04 | 0,08 | 0,00 | 0,40 | 0,00 | 0,14 |
| **FW68** | 0,00 | 0,00 | 0,00 | 0,00 | 0,00 | 0,00 | 0,00 | 0,00 | 0,00 | 0,00 | 0,00 |
| **iii1-15** | 0,00 | 0,06 | 0,00 | 0,00 | 0,00 | 0,00 | 0,00 | 0,00 | 0,00 | 0,00 | 0,00 |
| **Lactobacillales** | 0,00 | 3,66 | 0,00 | 0,00 | 2,85 | 0,00 | 0,00 | 0,00 | 0,00 | 0,00 | 0,00 |
| **Oceanospirillales** | 12,24 | 15,10 | 15,33 | 14,84 | 10,79 | 3,54 | 30,03 | 12,32 | 5,55 | 6,08 | 1,23 |
| **Neisseriales** | 0,00 | 0,00 | 0,00 | 0,00 | 0,00 | 0,00 | 0,00 | 0,00 | 0,50 | 0,00 | 0,00 |
| **Nostocales** | 0,00 | 0,00 | 0,00 | 0,00 | 0,00 | 0,00 | 0,00 | 0,00 | 0,00 | 0,00 | 0,00 |
| **Legionellales** | 0,00 | 0,00 | 0,00 | 0,00 | 0,00 | 0,00 | 0,00 | 0,00 | 0,00 | 0,00 | 0,00 |
| **Myxococcales** | 0,20 | 0,00 | 0,00 | 0,00 | 0,00 | 0,00 | 0,00 | 0,00 | 0,00 | 0,00 | 0,00 |
| **RB41** | 0,00 | 0,79 | 0,00 | 0,00 | 0,00 | 0,00 | 0,00 | 0,00 | 0,00 | 0,00 | 0,00 |
| **Pasteurellales** | 0,00 | 0,74 | 0,00 | 0,00 | 0,59 | 0,00 | 0,00 | 0,00 | 0,00 | 0,00 | 0,00 |
| **Pseudomonadales** | 18,13 | 22,46 | 18,68 | 34,52 | 17,49 | 2,98 | 12,79 | 20,98 | 22,73 | 9,45 | 19,47 |
| **Rhizobiales** | 1,10 | 0,00 | 0,18 | 0,00 | 0,00 | 1,14 | 0,00 | 5,98 | 0,00 | 0,00 | 0,00 |
| **Rhodobacterales** | 7,35 | 4,81 | 1,01 | 6,22 | 4,40 | 0,78 | 8,13 | 11,24 | 0,89 | 0,64 | 0,62 |
| **Rhodospirillales** | 0,00 | 0,00 | 0,00 | 0,00 | 0,00 | 0,00 | 0,00 | 0,00 | 0,00 | 0,00 | 0,00 |
| **Rickettsiales** | 0,00 | 0,00 | 0,00 | 0,00 | 0,00 | 0,00 | 0,00 | 0,00 | 0,00 | 0,00 | 0,00 |
| **Solibacterales** | 0,00 | 0,00 | 0,00 | 0,00 | 0,00 | 0,00 | 0,00 | 0,00 | 0,00 | 0,00 | 0,00 |
| **Solirubrobacterales** | 0,00 | 0,00 | 0,00 | 0,00 | 0,00 | 0,00 | 0,00 | 0,00 | 0,00 | 0,00 | 0,00 |
| **Sphingobacteriales** | 0,00 | 0,00 | 0,00 | 0,00 | 0,00 | 0,00 | 0,00 | 0,00 | 0,00 | 0,00 | 0,00 |
| **Sphingomonadales** | 0,13 | 0,00 | 3,03 | 0,00 | 0,00 | 0,00 | 0,00 | 0,00 | 1,02 | 0,00 | 0,00 |
| **Synechococcales** | 0,00 | 0,00 | 0,00 | 0,00 | 0,00 | 0,00 | 0,00 | 0,00 | 0,00 | 0,00 | 0,00 |
| **Thermogemmatisporales** | 0,00 | 0,00 | 0,00 | 0,00 | 0,00 | 0,00 | 0,00 | 0,00 | 0,00 | 0,00 | 0,00 |
| **Vibrionales** | 39,49 | 34,54 | 29,73 | 27,82 | 36,02 | 6,60 | 37,59 | 39,67 | 12,17 | 6,70 | 3,53 |
| **Xanthomonadales** | 0,00 | 0,00 | 0,91 | 5,24 | 0,01 | 0,00 | 0,66 | 0,00 | 0,00 | 0,00 | 0,00 |
| **ZA3648c** | 0,00 | 0,00 | 0,00 | 0,00 | 0,00 | 0,00 | 0,00 | 0,00 | 0,00 | 0,00 | 0,00 |
| **Unassigned** | 0,00 | 0,00 | 0,00 | 0,00 | 0,00 | 0,00 | 0,00 | 0,00 | 0,00 | 0,00 | 0,00 |

b)

| **Order/Samples** | ***M. alternatus*** | | | | | |
| --- | --- | --- | --- | --- | --- | --- |
|  | **Hikobe** | | | | | |
|  | **MA.JH55** | **MA.JH56** | **MA.JH59** | **MA.JH60** | **MA.JH61** | **MA.JH66** |
| **[Saprospirales]** | 0,00 | 2,48 | 0,36 | 0,49 | 0,00 | 0,02 |
| **Acidobacteriales** | 0,91 | 0,02 | 18,15 | 4,48 | 0,12 | 1,37 |
| **Acidimicrobiales** | 0,04 | 0,00 | 0,22 | 0,00 | 0,00 | 0,00 |
| **Actinomycetales** | 0,05 | 0,07 | 0,52 | 0,07 | 0,01 | 0,06 |
| **Aeromonadales** | 0,00 | 0,00 | 0,00 | 0,05 | 0,00 | 0,00 |
| **Alteromonadales** | 0,00 | 0,00 | 0,00 | 0,02 | 0,02 | 0,00 |
| **Arctic96B-7** | 0,15 | 0,00 | 0,00 | 0,04 | 0,01 | 0,00 |
| **Bdellovibrionales** | 0,00 | 0,00 | 0,00 | 0,04 | 0,00 | 0,00 |
| **Bacillales** | 2,16 | 0,00 | 0,00 | 0,79 | 0,00 | 0,06 |
| **Burkholderiales** | 3,09 | 0,16 | 5,50 | 1,13 | 0,09 | 12,02 |
| **Clostridiales** | 0,00 | 0,00 | 0,00 | 0,00 | 0,00 | 0,89 |
| **Caulobacterales** | 0,00 | 0,00 | 0,00 | 0,56 | 0,00 | 0,25 |
| **Cytophagales** | 0,00 | 0,00 | 0,00 | 0,20 | 0,00 | 0,00 |
| **Enterobacteriales** | 68,15 | 76,31 | 20,81 | 24,48 | 98,59 | 63,15 |
| **Elev-1554** | 0,00 | 0,00 | 0,00 | 0,04 | 0,00 | 0,00 |
| **Flavobacteriales** | 0,04 | 3,27 | 2,69 | 0,22 | 0,02 | 0,08 |
| **FW68** | 0,00 | 0,00 | 0,00 | 1,39 | 0,00 | 0,00 |
| **iii1-15** | 0,00 | 0,00 | 0,00 | 0,00 | 0,00 | 0,00 |
| **Lactobacillales** | 0,31 | 0,00 | 0,00 | 31,76 | 0,00 | 0,00 |
| **Oceanospirillales** | 2,53 | 0,01 | 2,69 | 0,65 | 0,24 | 3,48 |
| **Neisseriales** | 0,00 | 0,00 | 0,00 | 0,00 | 0,02 | 0,00 |
| **Nostocales** | 0,00 | 0,00 | 0,00 | 0,97 | 0,00 | 0,00 |
| **Legionellales** | 0,00 | 0,00 | 0,00 | 0,11 | 0,00 | 0,00 |
| **Myxococcales** | 0,00 | 0,00 | 0,24 | 0,00 | 0,00 | 0,32 |
| **RB41** | 0,00 | 0,00 | 0,00 | 0,00 | 0,00 | 0,00 |
| **Pasteurellales** | 0,00 | 0,00 | 0,00 | 0,00 | 0,00 | 0,00 |
| **Pseudomonadales** | 8,67 | 0,14 | 8,29 | 8,05 | 0,50 | 5,00 |
| **Rhizobiales** | 2,76 | 1,49 | 9,83 | 6,06 | 0,13 | 0,37 |
| **Rhodobacterales** | 1,14 | 0,00 | 0,73 | 0,37 | 0,01 | 1,55 |
| **Rhodospirillales** | 0,00 | 0,00 | 1,85 | 7,04 | 0,01 | 0,08 |
| **Rickettsiales** | 1,51 | 0,02 | 0,00 | 0,59 | 0,08 | 0,00 |
| **Solibacterales** | 0,00 | 0,00 | 0,00 | 1,71 | 0,00 | 0,00 |
| **Solirubrobacterales** | 0,04 | 0,00 | 0,00 | 0,00 | 0,00 | 0,00 |
| **Sphingobacteriales** | 0,00 | 0,09 | 5,17 | 0,34 | 0,00 | 2,14 |
| **Sphingomonadales** | 0,47 | 0,02 | 10,78 | 5,07 | 0,02 | 1,30 |
| **Synechococcales** | 0,45 | 0,01 | 0,00 | 0,05 | 0,01 | 0,00 |
| **Thermogemmatisporales** | 0,00 | 0,00 | 0,00 | 0,02 | 0,00 | 0,00 |
| **Vibrionales** | 6,03 | 0,04 | 3,15 | 1,77 | 0,14 | 6,22 |
| **Xanthomonadales** | 1,09 | 15,06 | 8,80 | 0,00 | 0,01 | 1,05 |
| **ZA3648c** | 0,00 | 0,00 | 0,00 | 0,04 | 0,00 | 0,00 |
| **Unassigned** | 0,42 | 0,82 | 0,19 | 1,40 | 0,00 | 0,59 |

Supplementary Figure S1 - Rarefaction curves of OTUs as function of the number of sequences.


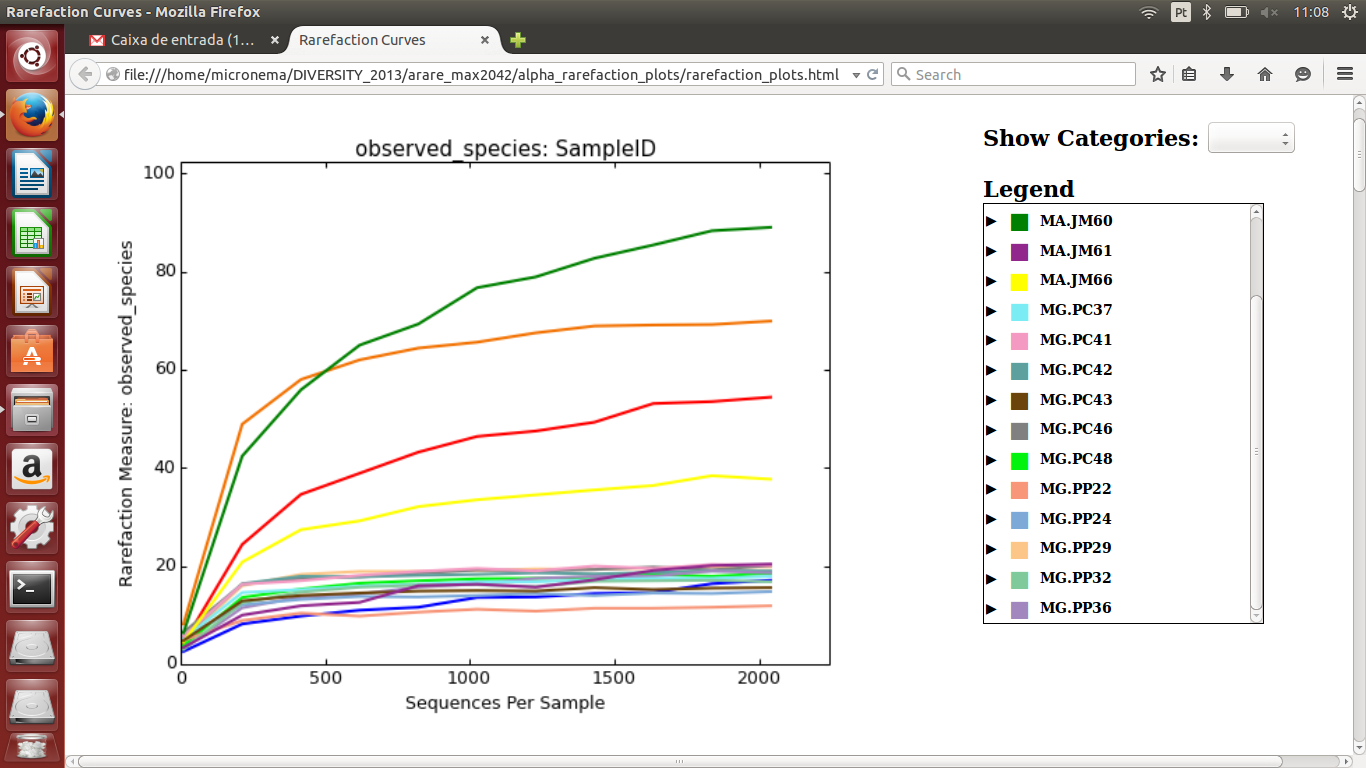

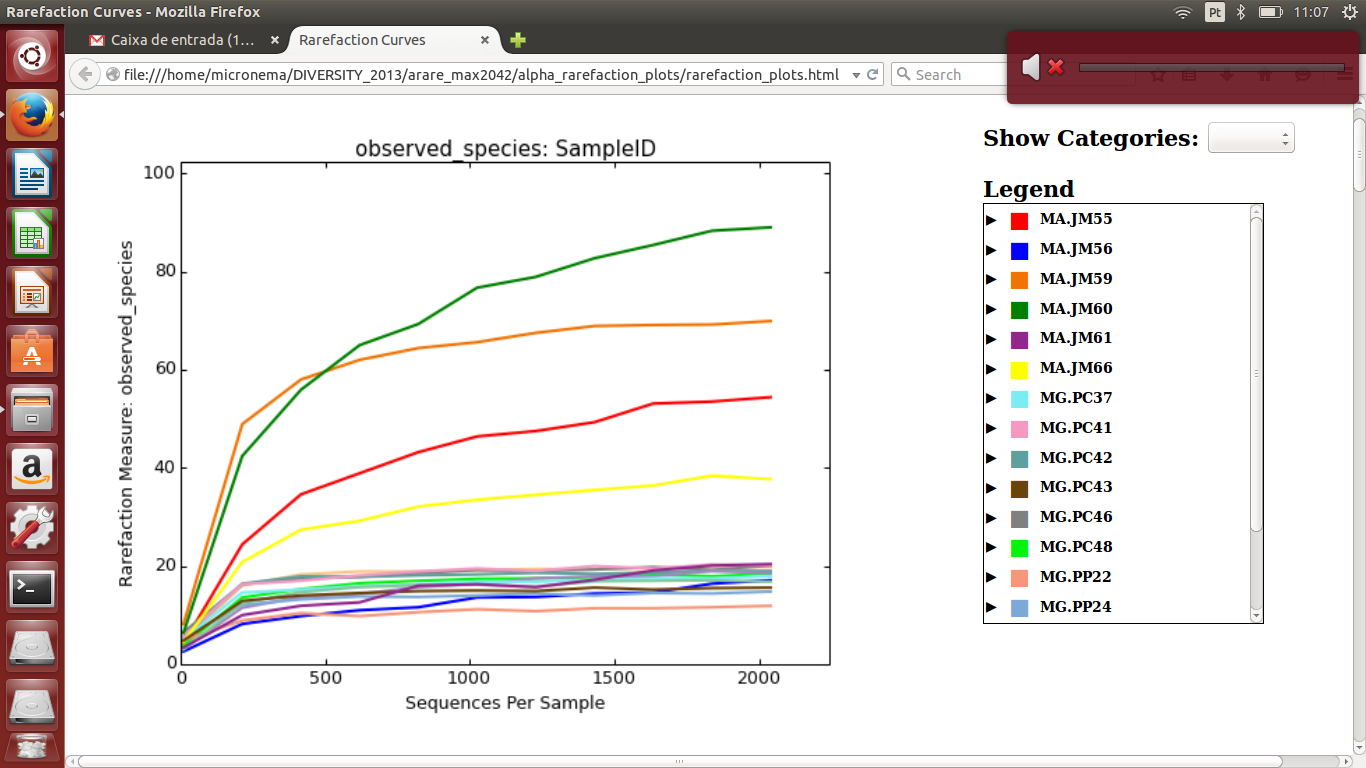


Sequences per sample


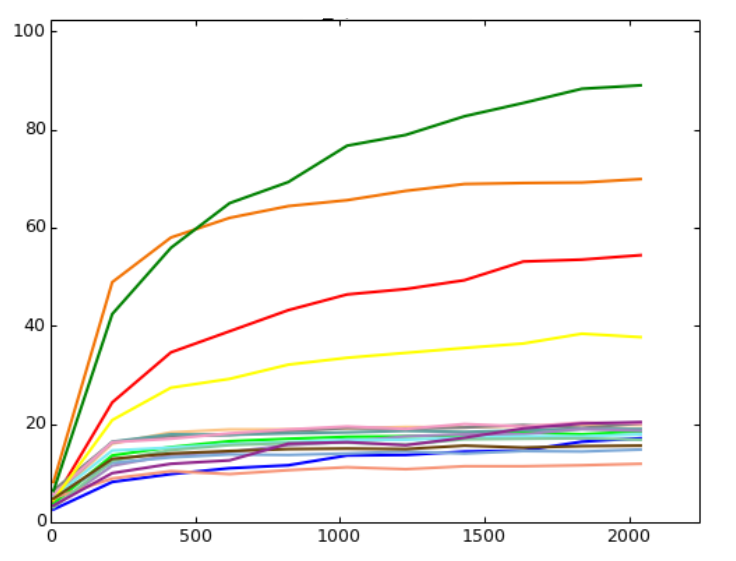


Observed OTUs
